# 6-Pyrazolylpurine and its deaza derivatives as nucleobases for silver(I)-mediated base pairing with pyrimidines

**DOI:** 10.1007/s00775-023-02022-0

**Published:** 2023-11-20

**Authors:** Daniela Escher, Tim Schäfer, Marian Hebenbrock, Jens Müller

**Affiliations:** 1 Institut für Anorganische und Analytische Chemie, Universität Münster, Corrensstr. 30, 48149 Münster, Germany; 2 Center for Soft Nanoscience (SoN) and Cells in Motion Interfaculty Centre (CiMIC), Universität Münster, Corrensstr. 30, 48149 Münster, Germany

**Keywords:** Cytosine, Metal-mediated base pair, Purine, Thymine

## Abstract

The artificial nucleobase 6-pyrazolylpurine (6PP) and its deaza derivatives 1-deaza-6-pyrazolylpurine (^1D^6PP), 7-deaza-6-pyrazolylpurine (^7D^6PP), and 1,7-dideaza-6-pyrazolylpurine (^1,7D^6PP) were investigated with respect to their ability to differentiate between the canonical nucleobases cytosine and thymine by means of silver(I)-mediated base pairing. As shown by temperature-dependent UV spectroscopy and by circular dichroism spectroscopy, 6PP and (to a lesser extent) ^7D^6PP form stable silver(I)-mediated base pairs with cytosine, but not with thymine. ^1D^6PP and ^1,7D^6PP do not engage in the formation of stabilizing silver(I)-mediated base pairs with cytosine or thymine. The different behavior of ^1D^6PP, ^7D^6PP, and ^1,7D^6PP indicates that silver(I) binding occurs via the N1 position of the purine derivative, i.e. via the Watson–Crick face. The data show that 6PP is capable of differentiating between cytosine and thymine, which is potentially relevant in the context of detecting single-nucleotide polymorphisms.

**Graphical abstract:**

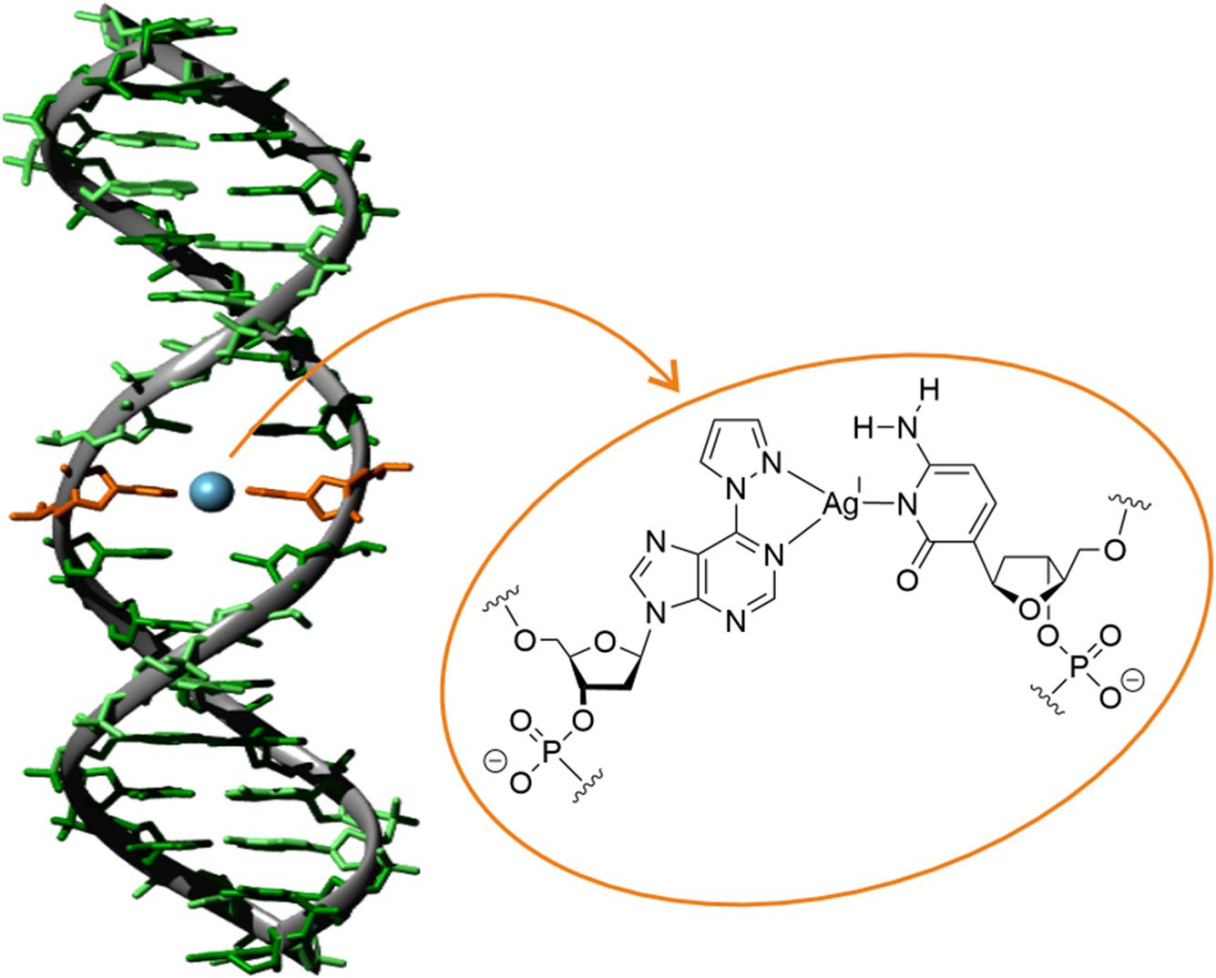

**Supplementary Information:**

The online version contains supplementary material available at 10.1007/s00775-023-02022-0.

## Introduction

Nucleic acids are highly versatile molecules with applications far beyond their original biological relevance. Synthetically modified nucleic acids are of particular interest in the context of supramolecular chemistry and nanotechnology [1]. The site-specific incorporation of transition metal ions into nucleic acids, i.e. the combination of evolutionary optimized self-assembly properties with metal-based functionality, broadens the scope of artificial nucleic acids significantly. It can be achieved by using metal-mediated base pairs, in which the hydrogen bonds between complementary nucleobases are formally replaced by coordinate bonds [2]. Towards this end, numerous artificial nucleobases have been developed [3, 4]. While the canonical nucleobases thymine and cytosine are known to form homoleptic metal-mediated base pairs with mercury(II) and silver(I) [5], the development of artificial metal-coordinating nucleobases allows the incorporation of other metal ions, too [6–10]. Likewise, the introduction of more than one metal ion per base pair can be achieved [11, 12]. Even arrays of metal ions within a DNA duplex [13–18] and organometallic DNA modifications are possible [9, 19–21]. DNA with metal-mediated base pairs can be applied to modulate the charge transfer ability of nucleic acids [22–25], to generate metal nanoclusters [26], to devise adaptive and responsive nanosystems [27–32], and to develop sensors for various analytes [33, 34]. The detection of oligonucleotide sequences via metal-mediated based pairing has raised particular interest [35]. Several artificial nucleobases have been investigated in this respect, including 1*H*-imidazo-[4,5-*f*][1,10]phenanthroline [36, 37], 3-fluoro-2-mercuri-6-methylaniline [38, 39], and 3,5-dimethylpyrazole-substituted purines [40].

**Fig. 1:**
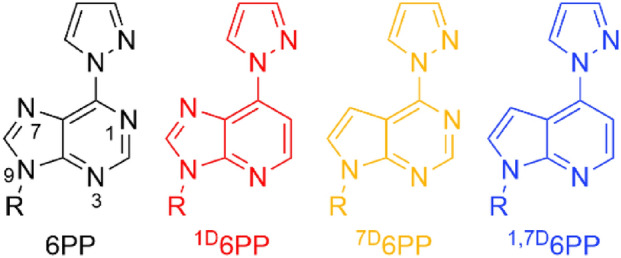
Chemical representation of 6PP (including atom numbering scheme of relevant endocyclic nitrogen atoms), ^1D^6PP, ^7D^6PP, and ^1,7D^6PP (R = H or 2’-deoxyribose), using the color scheme applied throughout this contribution to indicate the respective experimental data

We had been interested in whether the bulk of the 3,5-dimethylpyrazole substituent is relevant for the discrimination of the complementary nucleobase or whether the smaller pyrazole substituent would suffice and had therefore evaluated the metal-mediated base pairing ability of 6-pyrazolylpurine (6PP, Fig. 1) [41]. That study had indicated that a 6PP:C pair is slightly destabilized upon the addition of Ag(I), whereas a 6PP:T pair is marginally stabilized, indicating a discrimination of the pyrimidine residue even in the absence of methyl substituents on the pyrazole residue [41]. A subsequent extension of that work involved homo base pairs of 6PP as well as its deaza derivatives 1-deaza-6-pyrazolylpurine (^1D^6PP), 7-deaza-6-pyrazolylpurine (^7D^6PP), and 1,7-dideaza-6-pyrazolylpurine (^1,7D^6PP) (Fig. 1) [42, 43]. Those experiments identified the Watson–Crick edge of the purine derivative as the relevant silver(I)-binding site.

Unexpectedly, the deaza derivatives show different silver(I)-mediated base pairing properties with respect to complementary pyrimidine residues than the parent nucleobase 6PP (vide infra). This lead us to reinvestigate the silver(I)-binding ability of duplexes with 6PP:C and 6PP:T pairs. The newly obtained data are in good agreement with those of the deaza derivatives (vide infra) and contrast the published ones [41]. A thorough investigation of the previously reported results suggests that the artificial nucleobase in the 6PP-containing oligonucleotides must have decomposed or been chemically modified during or after solid-phase oligonucleotide synthesis without being tracked by the mass-spectrometric analysis. Unfortunately, attempts to identify the 6PP-derived nucleobase erroneously investigated in that study (by deliberately synthesizing and characterizing various possible decomposition products of similar molecular mass) remained unsuccessful (data not shown). To correct the chemical record, the following reports the results of the repeated experiments including those of the deaza derivatives. In this context, the syntheses and characterization data of the monomeric building blocks are in part reported again, indicating the reproducibility of the previously reported ones.

## Materials and methods

### General methods

All chemicals and solvents used for this project were purchased from ABCR, Acros Organics, Alfa Aesar, Carl Roth GmbH & Co. KG, Eurogentec, Fisher Scientific, GlenResearch, Merck, Sigma-Aldrich, TCI and VWR. If anhydrous solvents or reagents were required for synthesis, they were dried and distilled according to standard procedures. Only anhydrous pyridine was used as purchased from Acros Organics. Column chromatographic purifications were performed using Silica Gel 60 with a pore size of 60 Å and a particle size of 35–70 μm purchased from Acros Organics and Merck. The thin-layer chromatography plates DC Silica Gel 60 F254 were obtained from Merck. 6PP (**1**) and Hoffer’s chloro sugar were synthesized using previously published procedures [44, 45]. The elemental analysis was performed on a Vario EL III CHNS analyzer from Elementar Analysensysteme GmbH.

### Oligonucleotide synthesis and characterization

Phosphoramidites required for the synthesis of the oligonucleotide sequences were purchased from Glen Research. Syntheses of the oligonucleotide strands were performed on a K&A Laborgeräte H8 DNA/RNA synthesizer in the DMT-off mode by following standard protocols. Post-synthesis, the oligonucleotides were cleaved from the solid support and deprotected. For this purpose, a solution of aqueous ammonia (25%) and aqueous methylamine (40%) (1:1, v:v) was used for DNA comprising ^1D^6PP or ^7D^6PP nucleosides and a solution of ^*tert*^butylamine, aqueous ammonia (25%) and methanol (1:1:2, v:v:v) for those comprising 6PP or ^1,7D^6PP nucleosides. If the former solution was used, the deprotection was performed at 65 °C for 15 min, and for the latter, the temperature was adjusted to 55 °C and the duration extended to 3 h. Thereafter, the oligonucleotides were purified by denaturing urea polyacrylamide gel electrophoresis (gel solution: 7 M urea, 1 M TBE, 18% polyacrylamide:bisacrylamide (29:1); running buffer: 0.1 M TBE; loading buffer: 11.8 M urea, 42 mM Tris/HCl (pH 7.5), 0.83 mM EDTA (pH 8.0), 8% sucrose, 0.08% dye (xylene cyanol, bromophenol blue)). Afterwards, the DNA was extracted from the gels by electroelution in TBE buffer (0.1 M) and desalted twice with NAP 10 columns. The desalted oligonucleotides were characterized by MALDI-TOF mass spectrometry. MALDI-TOF mass spectra were recorded on either a Reflex IV MALDI-TOF or an Autoflex Speed MALDI-TOF spectrometer (Bruker) using a 3-hydroxypicolinic acid/ammonium citrate matrix. During the quantification of the oligonucleotides, the following molar absorption coefficients *ε*_260_ were used: 6PP, 4.8 cm^2^ μmol^–1^; ^1D^6PP, 8.6 cm^2^ μmol^–1^; ^7D^6PP, 7.0 cm^2^ μmol^–1^; ^1,7D^6PP, 2.5 cm^2^ μmol^–1^ [42].

### Spectroscopy

NMR spectra were recorded using Bruker Avance(I) 400 and Bruker Avance(III) 400 spectrometers. The samples under investigation were dissolved in deuterated solvents purchased from Acros Organics or Sigma-Aldrich. For the assignment of the chemical shifts observed in the ^1^H and ^13^C{^1^H} NMR spectra, the residual solvent signals were used. In addition, tetramethylsilane (TMS) was used for studies in CDCl_3_ and sodium 3-(trimethylsilyl)-1-propanesulfonate (TSP) for studies in D_2_O. ^31^P{^1^H} NMR spectra were referenced using orthophosphoric acid (*c* = 85%). The data were evaluated using the software MestReNova (Mestrelab Research S. L.). DNA melting experiments were carried out on a UV spectrometer CARY 100 Bio using a 1 cm quartz cuvette. The UV melting profiles were measured at 260 nm in buffer at pH 6.8 (1 μM oligonucleotide duplex, 150 mM NaClO_4_, 5 mM MOPS (pH 6.8) or 5 mM borate (pH 9.0)) in the absence or presence of AgNO_3_, at a heating rate of 1 °C min^–1^ with data being recorded at an interval of 1 °C. Melting temperatures were determined from the maxima of the first derivatives of the melting curves. CD spectra were recorded at 5 °C on a J-815 CD spectrometer using the same samples as for the UV melting profiles.

### Syntheses

6-(1*H*-Pyrazol-1-yl)-9*H*-purine (6PP) (**1**) [41, 44].

6-Chloropurine (1.00 g, 6.48 mmol) and pyrazole (2.20 g, 32.3 mmol) were mixed and stirred at 150 °C for 1 h. After cooling, DCM (5 mL) was added to the crude product and the mixture was stirred until a uniform slurry was obtained. Afterwards, the mixture was filtered, and the residue was washed with DCM (5 × 5 mL) and Et_2_O (3 × 10 mL) to obtain compound **1** as a light brown powder. Yield: 1.14 g (6.14 mmol, 95%). ^1^H NMR (400 MHz, DMSO-*d*_6_): *δ* (ppm) = 13.17 (s, br, NH), 8.90 (s, 1H, H5*), 8.82 (s, 1H, H2), 8.66 (s, 1H, H8), 8.09 (d, ^3^*J*_HH_ = 1.7 Hz, 1H, H3*), 6.75 (t, ^3^*J*_HH_ = 2.7 Hz, ^3^*J*_HH_ = 1.6 Hz, 1H, H4*). (* refers to pyrazole atoms).

9-(2-Deoxy-3,5-di-*O*-p-toluoyl-β-d-erythropentofuranosyl)-6-(1*H*-pyrazol-1-yl)-9*H*-purine (**2**) [41].

6PP (**1**, 480 mg, 2.58 mmol) was suspended in dry MeCN (20 mL). NaH (60% dispersion in mineral oil, 170 mg, 4.25 mmol) was added at 0 °C and the mixture was stirred for 1 h at room temperature. Hoffer’s chloro sugar [45] (1.25 g, 3.22 mmol) was suspended in toluene (20 mL) and the suspension was added in four steps within 20 min. The mixture was stirred for 20 h at room temperature. After purification by column chromatography (Cy (8):EtOAc (4):Et_3_N (3):DCM (1); Cy = cyclohexane) and recrystallization from EtOAc, compound **2** was obtained as white, acicular crystals. Yield: 702 mg (1.30 mmol, 51%). ^1^H NMR (400 MHz, CDCl_3_): *δ* (ppm) = 9.01 (d, ^3^*J*_HH_ = 2.7 Hz, 1H, H5*), 8.76 (s, 1H, H2), 8.30 (s, 1H, H8), 8.01 – 7.95 (m, 3H, H3*, H_ortho_), 7.92 – 7.84 (m, 2H, H_ortho_), 7.29 (d, ^3^*J*_HH_ = 8.0 Hz, 2H, H_meta_), 7.19 (d, ^3^*J*_HH_ = 8.0 Hz, 2H, H_meta_), 6.64 (dd, ^3^*J*_HH_ = 8.2 Hz, ^3^*J*_HH_ = 5.8 Hz, 1H, H1'), 6.57 (dd, ^3^*J*_HH_ = 2.8 Hz, ^3^*J*_HH_ = 1.6 Hz, 1H, H4*), 5.85 (dt, ^3^*J*_HH_ = 6.4 Hz, ^3^*J*_HH_ = 2.2 Hz, 1H, H3'), 4.80 (dd, ^2^*J*_HH_ = 13.3 Hz, ^3^*J*_HH_ = 5.1 Hz, 1H, H5' or H5''), 4.71 – 4.61 (m, 2H, H5' or H5'', H4'), 3.18 (ddd, ^2^*J*_HH_ = 14.4 Hz, ^3^*J*_HH_ = 8.3 Hz, ^3^*J*_HH_ = 6.3 Hz, 1H, H2' or H2''), 2.90 (ddd, ^2^*J*_HH_ = 14.1 Hz, ^3^*J*_HH_ = 5.8 Hz, ^3^*J*_HH_ = 2.2 Hz, 1H, H2' or H2''), 2.45 (s, 3H, CH_3_), 2.37 (s, 3H, CH_3_). (* refers to pyrazole atoms).

9-(2-Deoxy-β-d-erythropentofuranosyl)-6-(1*H*-pyrazol-1-yl)-9*H*-purine (**3**) [41].

Compound **2** (749 mg, 1.39 mmol) was stirred in a mixture of aqueous ammonia (25% in H_2_O, 50 mL) and MeOH (50 mL) for 4 h at room temperature. The solvent was removed at 0 °C under vacuum and the residue was purified by column chromatography (Cy (8):EtOAc (5): DCM (3): MeOH (1)). A white solid of **3** was obtained. Yield: 294 mg (0.973 mmol, 70%). ^1^H NMR (400 MHz, MeOD): *δ* (ppm) = 9.11 (d, ^3^*J*_HH_ = 2.7 Hz, 1H, H5*), 8.74 (s, 1H, H2), 8.72 (s, 1H, H8), 7.95 (d, ^3^*J*_HH_ = 1.7 Hz, 1H, H3*), 6.66 (dd, ^3^*J*_HH_ = 2.9 Hz, 1.5 Hz, 1H, H4*), 6.58 (t, ^3^*J*_HH_ = 6.7 Hz, 1H, H1'), 4.62 (dt, ^3^*J*_HH_ = 6.4 Hz, ^3^*J*_HH_ = 3.4 Hz, 1H, H3'), 4.08 (q, ^3^*J*_HH_ = 3.5 Hz, 1H, H4'), 3.85 (dd, ^2^*J*_HH_ = 12.1 Hz, ^3^*J*_HH_ = 3.5 Hz, 1H, H5' or H5''), 3.77 (dd, ^2^*J*_HH_ = 12.1 Hz, ^3^*J*_HH_ = 4.0 Hz, 1H, H5' or H5''), 2.86 (ddd, ^2^*J*_HH_ = 13.3 Hz, ^3^*J*_HH_ = 7.2 Hz, ^3^*J*_HH_ = 6.0 Hz, 1H, H2' or H2''), 2.52 (ddd, ^2^*J*_HH_ = 13.6 Hz, ^3^*J*_HH_ = 6.3 Hz, ^3^*J*_HH_ = 3.5 Hz, 1H, H2' or H2''). ^13^C{^1^H} NMR (101 MHz, MeOD): *δ* (ppm) = 154.6 (C4), 152.8 (C2), 148.1 (C6), 146.1 (C8), 145.3 (C3*), 132.5 (C5*), 123.7 (C5), 110.3 (C4*), 89.7 (C4'), 86.7 (C1'), 72.7 (C3'), 63.3 (C5'), 41.5 (C2'). (* refers to pyrazole atoms).

9-[2-Deoxy-5-*O*-(4,4’-dimethoxytrityl)-β-d-erythropentofuranosyl]-6-(1*H*-pyrazol-1-yl)-9*H*-purine (**4**) [41].

The 6PP nucleoside **3** (326 mg, 1.08 mmol) was dissolved in anhydrous pyridine (5 mL). A solution of DMT-Cl (455 mg, 1.34 mmol) in anhydrous pyridine (5 mL) was then added dropwise to the former and the obtained yellow solution was stirred for 3 h at room temperature. The solvent was removed and the crude product was purified by column chromatography (DCM (20):EtOAc (4):MeOH (1)) to obtain **4** as a white solid foam. Yield: 402 mg (0.665 mmol, 62%). ^1^H NMR (400 MHz, CD_2_Cl_2_): *δ* (ppm) = 9.11 (dd, ^3^*J*_HH_ = 2.8 Hz, ^4^*J*_HH_ = 0.7 Hz, 1H, H5*), 8.71 (s, 1H, H2), 8.27 (s, 1H, H8), 7.94 – 7.87 (m, 1H, H3*), 7.44 – 7.39 (m, 2H, H_ortho_), 7.32 – 7.28 (m, 4H, H_ortho_), 7.28 – 7.24 (m, 2H, H_meta_), 7.23 – 7.18 (m, 1H, H_para_), 6.80 (dq, ^3^*J*_HH_ = 8.5 Hz, ^3^*J*_HH_ = 3.1 Hz, 4H, H_meta_), 6.59 (dd, ^3^*J*_HH_ = 2.8 Hz, ^3^*J*_HH_ = 1.5 Hz, 1H, H4*), 6.53 (t, ^3^*J*_HH_ = 6.5 Hz, 1H, H1'), 4.73 (q, ^3^*J*_HH_ = 4.7 Hz, 1H, H3'), 4.19 (q, ^3^*J*_HH_ = 4.7 Hz, 1H, H4'), 3.75 (s, 3H, OCH_3_), 3.75 (s, 3H, OCH_3_), 3.48 – 3.32 (m, 2H, H5' and H5''), 2.89 (dt, ^2^*J*_HH_ = 13.7 Hz, ^3^*J*_HH_ = 6.4 Hz, 1H, H2' or H2''), 2.58 (ddd, ^2^*J*_HH_ = 13.6 Hz, ^3^*J*_HH_ = 6.5 Hz, ^3^*J*_HH_ = 4.2 Hz, 1H, H2' or H2''). ^13^C{^1^H} NMR (101 MHz, CD_2_Cl_2_): *δ* (ppm) = 159.1 (C_q_OCH_3_), 153.8 (C4), 152.3 (C2), 147.7 (C6), 145.2 (C_q_), 144.4 (C3*), 143.6 (C8), 136.1 (C_q_), 131.7 (C5*), 130.4 (C_ortho_), 128.5 (C_ortho_), 128.3 (C_meta_), 127.3 (C_para_), 123.3 (C5), 113.5 (C_meta_), 109.3 (C4*), 87.0 (C4'), 86.8 (C_q_OC5’), 85.2 (C1'), 72.8 (C3'), 64.5 (C5'), 55.6 (OCH_3_), 40.7 (C2'). (* refers to pyrazole atoms).

9-[2-Deoxy-5-*O*-(4,4’-dimethoxytrityl)-β-d-erythropentofuranosyl]-6-(1*H*-pyrazol-1-yl)-9*H*-purine 3'-(2-cyanoethyl)-*N*,*N*-diisopropyl phosphoramidite (**5**) [41].

A solution of compound **4** (0.36 g, 0.59 mmol) in anhydrous DCM (5 mL) was treated with DIPEA (0.52 mL, 0.38 g, 3.0 mmol) and 2-cyanoethyl-*N*,*N*-diisopropylchlorophosphoramidite (CEDIP-Cl) (0.16 mL, 0.17 g, 0.71 mmol). The reaction mixture was stirred at room temperature for 15 min before the solvent was removed. The yellow oil was purified by column chromatography (Cy (1):EtOAc (3):Et_3_N (0.03)) to obtain a colorless oil of compound **5**. Yield: 0.23 g (0.28 mmol, 78%). ^1^H NMR (400 MHz, CD_3_CN): *δ* (ppm) = 9.11 (dd, ^3^*J*_HH_ = 2.7 Hz, ^4^*J*_HH_ = 0.8 Hz, 1H, H5*), 8.69 (s, 1H, H2), 8.40 (s, 1H, H8), 7.90 (dd, ^3^*J*_HH_ = 1.7 Hz, ^4^*J*_HH_ = 0.6 Hz, 1H, H3*), 7.39 – 7.34 (m, 2H, H_ortho_), 7.27 – 7.20 (m, 6H, H_ortho_, H_meta_), 7.19 – 7.14 (m, 1H, H_para_), 6.80 – 6.72 (m, 4H, H_meta_), 6.62 (dd, ^3^*J*_HH_ = 2.8 Hz, ^3^*J*_HH_ = 1.6 Hz, 1H, H4*), 6.49 (dd, ^3^*J*_HH_ = 7.0 Hz, ^3^*J*_HH_ = 5.5 Hz, 1H, H1'), 4.92 (ddt, ^2^*J*_HH_ = 11.1 Hz, ^3^*J*_HH_ = 6.7 Hz, ^3^*J*_HH_ = 4.9 Hz, 1H, H3'), 4.26 (q, ^3^*J*_HH_ = 4.1 Hz, 1H, H4'), 3.75 – 3.68 (m, 2H, OCH_2_), 3.71 (s, 3H, OCH_3_), 3.70 (s, 3H, OCH_3_), 3.62 (dq, ^3^*J*_HP_ = 10.3 Hz, ^3^*J*_HH_ = 6.7 Hz, 2H, ^*i*^Pr-CH), 3.37 (dd, ^2^*J*_HH_ = 10.6 Hz, ^3^*J*_HH_ = 3.6 Hz, 1H, H5' or H5''), 3.31 (dd, ^2^*J*_HH_ = 10.5 Hz, ^3^*J*_HH_ = 5.4 Hz, 1H, H5' or H5''), 3.09 (ddd, ^2^*J*_HH_ = 13.8 Hz, ^3^*J*_HH_ = 6.7 Hz, ^3^*J*_HH_ = 5.4 Hz, 1H, H2' or H2''), 2.63 (ddd, ^2^*J*_HH_ = 13.6 Hz, ^3^*J*_HH_ = 7.0 Hz, ^3^*J*_HH_ = 5.0 Hz, 1H, H2' or H2''), 2.55 (t, ^3^*J*_HH_ = 6.0 Hz, 2H, CH_2_CN), 1.19 (d, ^3^*J*_HH_ = 2.7 Hz, 6H, ^*i*^Pr-CH_3_), 1.17 (d, ^3^*J*_HH_ = 2.4 Hz, 6H, ^*i*^Pr-CH_3_). ^31^P{^1^H} NMR (162 MHz, CD_3_CN): *δ* (ppm) = 148.2, 148.1. (* refers to pyrazole atoms) MS (ESI): *m*/*z* = 827.3408 ([**5** + Na]^+^, calcd. *m*/*z* = 827.3405). Elemental analysis (C_43_H_49_N_8_O_6_P): C 63.8 (calcd. 64.2), H 5.9 (calcd. 6.1), N 13.5 (calcd. 13.9).

### Computational methods

DFT calculations were performed by the Gaussian 16 package [46]. Based on the CAM-B3LYP hybrid exchange–correlation functional [47] and SDD basis set [48], the Kohn–Sham molecular orbitals were calculated. Empirical dispersion was attributed by Grimme’s dispersion (version D3) with Becke-Johnson damping (BJ) [49]. The solvent (water) was taken into account via the Polarizable Continuum Model (PCM) in an integral equation formalism framework [50]. To evaluate the differences between the metal-binding abilities of ^7D^6PP:C and 6PP:C base pairs, the S_0_ geometry of each base pair with and without metal ions was optimized and their energies compared. The energy difference of the base pair with and without metal ion for ^7D^6PP was compared to the energy difference of the base pair with and without metal ion for 6PP. The difference of both values was used as the overall stabilization of 6PP:C compared to ^7D^6PP:C upon Ag(I) binding. Calculations with explicit water molecules were performed on the same theoretical level as mentioned above. Several starting geometries were utilized, and stable representative conformations were picked (Supplementary Material). The energetic minimum of each final geometry was confirmed by a frequency analysis. Thermochemical values were calculated at 298.15 K. Angles between the two nucleobase heterocycles were calculated with Mercury (CCDC) [51] and the structures visualized by CYLview [52].

## Results and discussion

### Synthesis

**Scheme 1:**
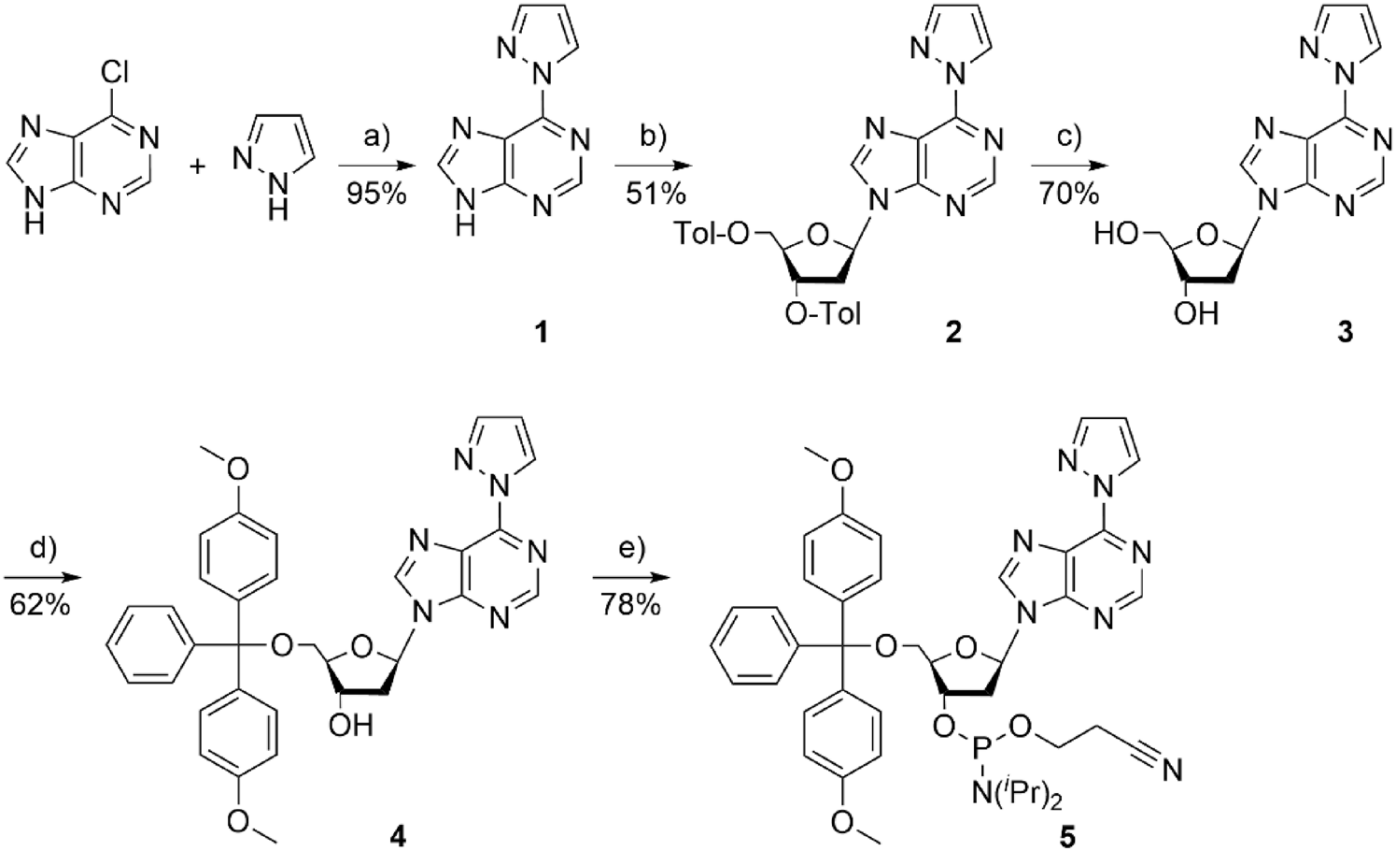
Synthesis of the 6-pyrazolylpurine (6PP) deoxyribonucleoside (**3**) and the corresponding phosphoramidite (**5**). **a** Neat, 150 °C, 1 h; **b** first step: NaH (1.6 equiv.), CH_3_CN, 0 °C, 1 h, second step: Hoffer’s chloro sugar (1.2 equiv.) [45], toluene, 20 h; **c** aqueous NH_3_ (25%), methanol, RT, 4 h; **d** DMT-Cl (1.2 equiv.), pyridine, RT, 3 h; **e** CEDIP-Cl (1.2 equiv.), DIPEA (5 equiv.), DCM, RT, 15 min

The artificial nucleosides were synthesized from pyrazole and the respective 6-chloropurine derivative as reported earlier [41, 42]. In the following, the synthesis of the phosphoramidite required for automated DNA synthesis is described exemplarily for 6PP (Scheme 1). For further details, see Materials and Methods section.

Initially, 6-chloropurine was stirred in neat pyrazole at 150 °C to give the free artificial nucleobase 6PP (**1**) [44]. Subsequent reaction with Hoffer’s chloro sugar [45] resulted in the formation of the *p*-toluoyl-protected β-configured 2’-deoxyribose **2**. The free deoxyribonucleoside **3** was obtained upon deprotection using aqueous ammonia. Its reaction with DMT-Cl gave compound **4**, which upon reaction with CEDIP-Cl was converted to the phosphoramidite **5**. This phosphoramidite was used for the subsequent automated solid-phase oligonucleotide synthesis. The respective deaza derivatives were synthesized analogously [42].

**Fig. 2:**
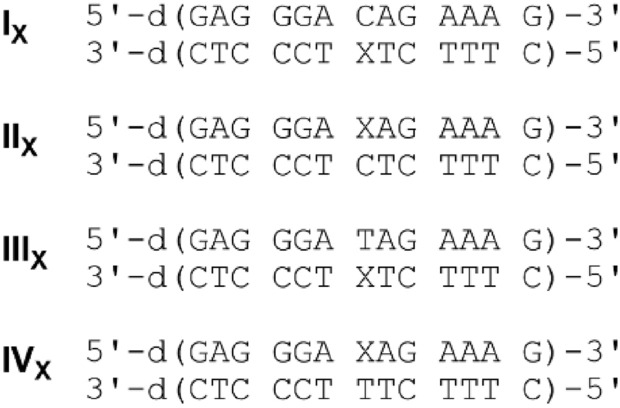
DNA oligonucleotide sequences used in this study, including their numbering scheme (X = 6PP, ^1D^6PP, ^7D^6PP, or ^1,7D^6PP)

The potential X:C and X:T base pairs (X = 6PP, ^1D^6PP, ^7D^6PP, or ^1,7D^6PP) were investigated in two closely related DNA duplex contexts each. The general duplex sequences are given in Fig. 2. They were chosen because several other metal-mediated base pairs have been reported in the same sequence context, allowing a comparison of the results [4]. In summary, 16 duplexes were investigated (**I**_**X**_ – **IV**_**X**_ with X = 6PP, ^1D^6PP, ^7D^6PP, ^1,7D^6PP). The Supplementary Material includes the mass-spectrometric characterization of these oligonucleotides.

Because of the previous problems with the decomposition / chemical modification of the artificial nucleoside 6PP during solid-phase synthesis (e.g. similar to what had been reported for the structurally related 2,6-bis(3,5-dimethylpyrazol-1-yl)purine [53]), mild deprotection conditions were used for oligonucleotides containing 6PP and ^1,7D^6PP (^*tert*^butylamine, aq. ammonia (25%), methanol (1:1:2), 55 °C, 3 h). In contrast, regular fast deprotection conditions turned out to be applicable when the oligonucleotides contained ^1D^6PP or ^7D^6PP without any decomposition (aq. ammonia (25%), aq. methylamine (40%) (1:1), 65 °C, 15 min).

### Spectroscopic characterization of the heteroleptic base pairs with cytosine

The silver(I)-binding behavior of duplexes **I**_**X**_ and **II**_**X**_ was investigated by temperature-dependent UV spectroscopy and by CD spectroscopy. Because of essentially identical trends, the results obtained for duplexes **I**_**X**_ will be discussed here in detail. For the respective data of duplexes **II**_**X**_, please see Supplementary Material (Fig. S1 and S2).

**Fig. 3:**
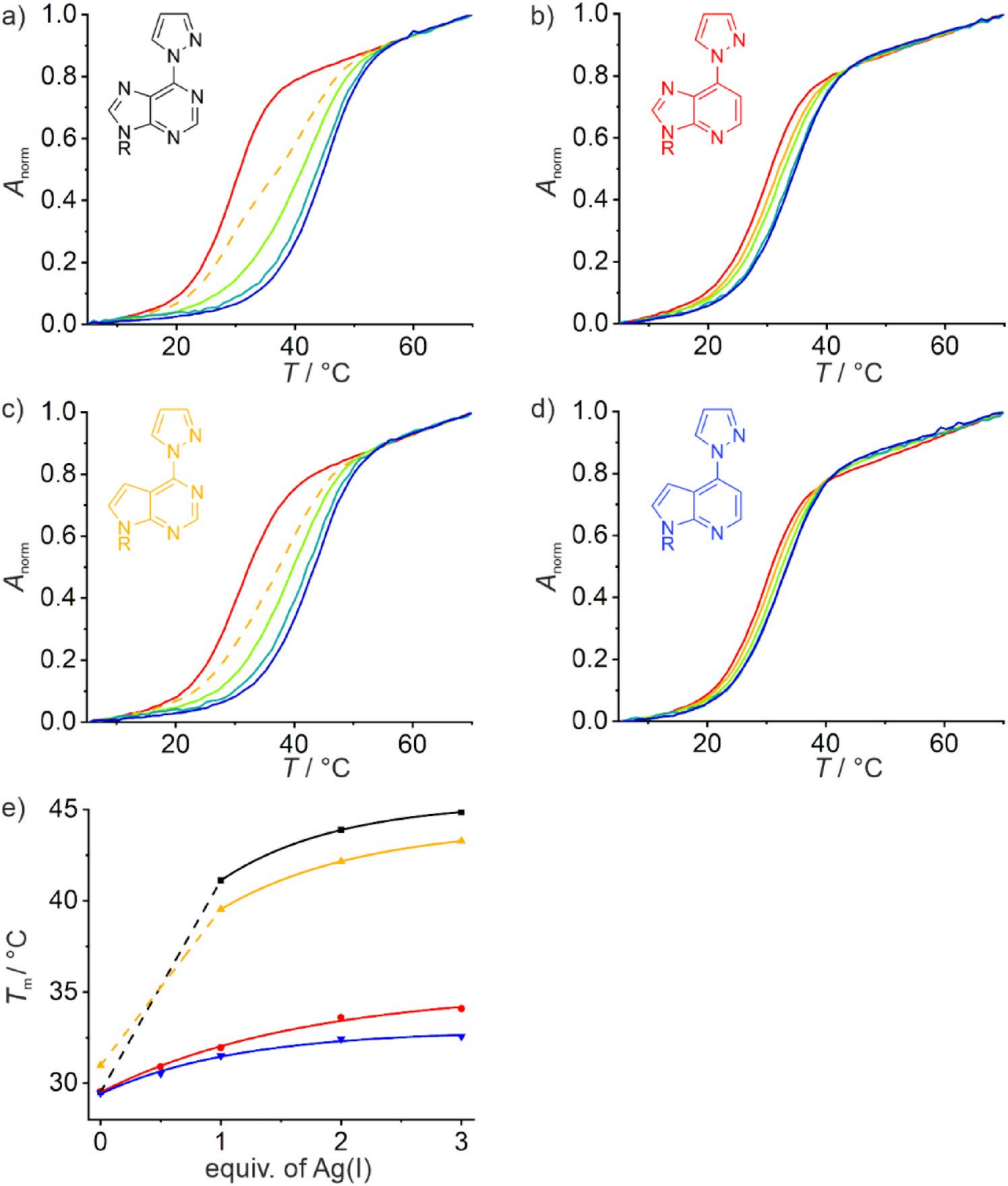
Melting curves of **a** duplex **I**_**6PP**_, **b** duplex ${\textbf{I}}_{{}^{{\textbf{1D}}}{\textbf{6PP}}}$, **c** duplex ${\textbf{I}}_{{}^{{\textbf{7D}}}{\textbf{6PP}}}$, and **d** duplex ${\textbf{I}}_{{}^{{\textbf{1,7D}}}{\textbf{6PP}}}$ with a cytosine residue located opposite X. Color code: red, no Ag(I); yellow: 0.5 equiv. of Ag(I); green: 1 equiv. of Ag(I); turquoise: 2 equiv. of Ag(I); blue: 3 equiv. of Ag(I). A melting curve plotted as a broken line indicates biphasic melting behavior. **e** Overview of the melting temperatures *T*_m_ of these duplexes depending on the amount of Ag(I). Broken lines indicate biphasic melting, hence no melting temperature can be determined in this region. Chemical representations of the nucleobases X and the melting temperatures of their corresponding duplexes **I**_**X**_ are shown in the same color. Conditions: 1 μM duplex, 5 mM MOPS (pH 6.8), 150 mM NaClO_4_

Figure 3 shows the melting curves of duplexes **I**_**X**_ in the absence and presence of different equivalents of silver(I). It also gives an overview of the melting temperatures *T*_m_ derived from these melting curves. The melting temperature of duplex **I**_**6PP**_ (Fig. 3a) increases significantly upon the addition of one equivalent of Ag(I) (Δ*T*_m_ = 11 °C), whereas excess Ag(I) leads to a minor additional increase only. Such a behavior is indicative of the formation of a metal-mediated base pair [54]. It is interesting to note that in the presence of substoichiometric amounts of Ag(I) (here: 0.5 equiv.) a biphasic melting transition is observed. When only one designated silver(I)-binding site is available as in duplex **I**_**6PP**_, biphasic melting is indicative of the formation of a kinetically inert metal complex, resulting in one part of the duplexes being silver(I)-free, whereas the other part contains a 6PP–Ag(I)–C base pair. The fact that the melting curves are monophasic again in the presence of excess Ag(I) confirms the formation of a metal-mediated base pair, as excess Ag(I) is expected to bind non-specifically and with lower affinity to the canonical nucleobases. A similar behavior is observed for duplex ${\textbf{I}}_{{}^{{\textbf{7D}}}{\textbf{6PP}}}$ (Fig. 3c), allowing to draw the conclusion that an analogous ^7D^6PP–Ag(I)–C base pair is formed. This base pair is of slightly lower stability (Δ*T*_m_ = 8 °C). Duplexes ${\textbf{I}}_{{}^{{\textbf{1D}}}{\textbf{6PP}}}$ (Fig. 3b) and ${\textbf{I}}_{{}^{{\textbf{1,7D}}}{\textbf{6PP}}}$ (Fig. 3d), both of which contain a 6PP derivative lacking the endocyclic N1 nitrogen atom, are not significantly stabilized upon the addition of Ag(I). In fact, they only show a minor increase in *T*_m_ that can be fitted asymptotically. This is indicative of non-specific binding of Ag(I), probably to the canonical nucleobases in the duplexes. Analogous observations are made for duplexes **II**_X_ (Fig. S1).

**Fig. 4:**
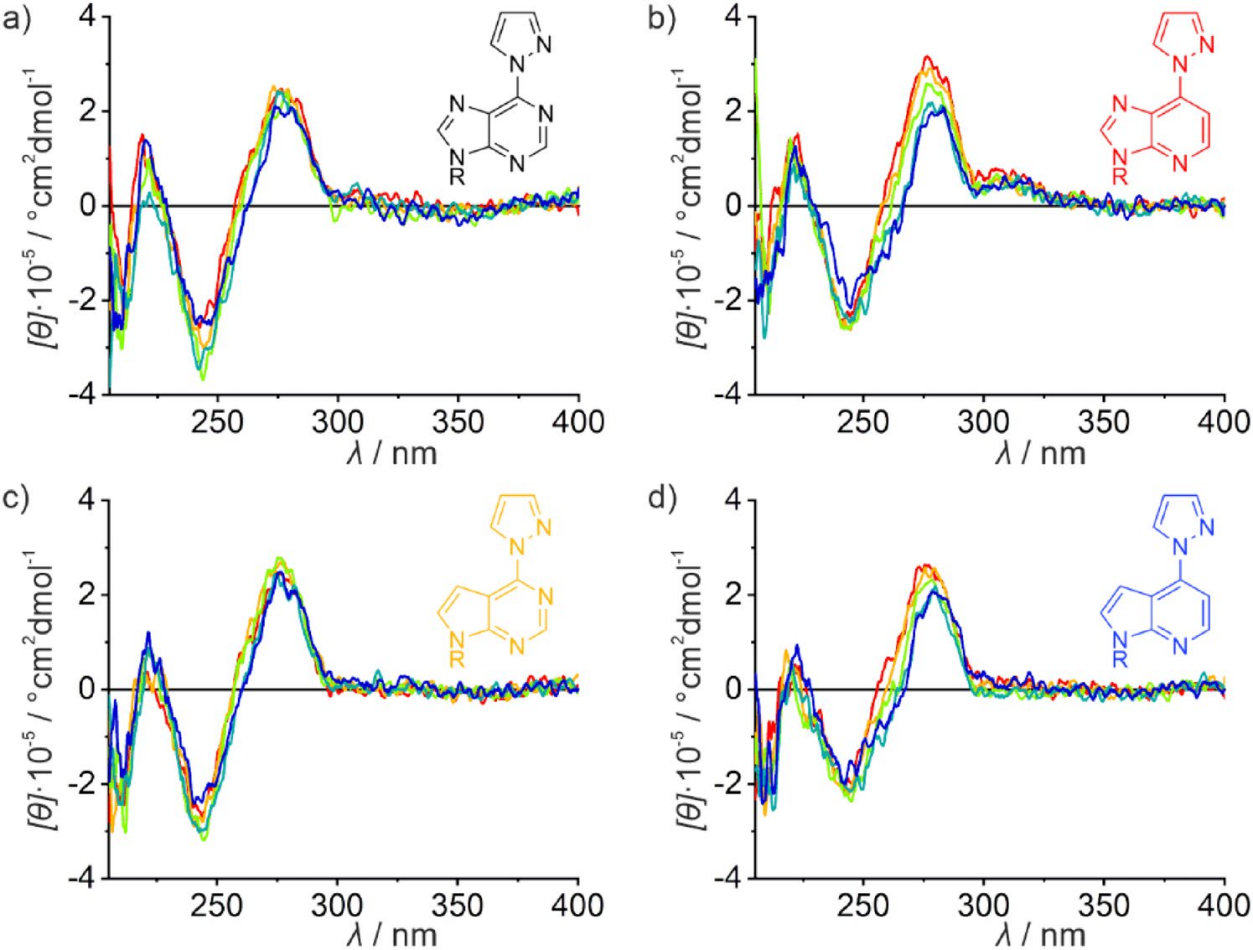
CD spectra of **a** duplex **I**_**6PP**_, **b** duplex ${\textbf{I}}_{{}^{{\textbf{1D}}}{\textbf{6PP}}}$, **c** duplex ${\textbf{I}}_{{}^{{\textbf{7D}}}{\textbf{6PP}}}$, and **d** duplex ${\textbf{I}}_{{}^{{\textbf{1,7D}}}{\textbf{6PP}}}$ with a cytosine residue located opposite X in the presence of increasing amounts of Ag(I). Color code: red, no Ag(I); yellow: 0.5 equiv. of Ag(I); green: 1 equiv. of Ag(I); turquoise: 2 equiv. of Ag(I); blue: 3 equiv. of Ag(I). Conditions: 1 μM duplex, 5 mM MOPS (pH 6.8), 150 mM NaClO_4_, 5 ℃

The CD spectra recorded during the titration of duplexes **I**_**X**_ with Ag(I) are summarized in Fig. 4. In the absence of silver(I), they all essentially resemble the CD spectrum of regular B-DNA, with a negative Cotton effect at ca. 245 nm and a positive one at around 280 nm [55]. Upon the addition of Ag(I), there are hardly any changes in the case of duplexes **I**_**6PP**_ (Fig. 4a) and ${\textbf{I}}_{{}^{{\textbf{7D}}}{\textbf{6PP}}}$ (Fig. 4c). In contrast, a decrease in intensity of the positive Cotton effect is observed for duplexes ${\textbf{I}}_{{}^{{\textbf{1D}}}{\textbf{6PP}}}$ (Fig. 4b) and ${\textbf{I}}_{{}^{{\textbf{1,7D}}}{\textbf{6PP}}}$ (Fig. 4d), accompanied by a slight red-shift of the Cotton effect. Such a change in the CD spectrum had been reported before for duplexes containing non-Watson–Crick-type Ag(I)-mediated base pairs involving canonical nucleobases [15, 56, 57]. It may therefore be considered further indication for the non-specific binding of the excess silver(I) to the duplexes under investigation. As the stability of the resulting non-specific DNA-silver(I) adducts is dependent on the exact oligonucleotide sequence, their formation (accompanied by the changes in the CD spectra) takes place at different silver(I) concentrations. Essentially the same observations are made for duplexes **II**_X_ (Fig. S2).

### Spectroscopic characterization of the heteroleptic base pairs with thymine

To evaluate the silver(I)-binding ability of the corresponding duplexes bearing a central X:T pair, duplexes **III**_**X**_ and **IV**_**X**_ underwent the same characterization as described above for duplexes **I**_**X**_ and **II**_**X**_. Again, the data obtained for duplexes **III**_**X**_ will be discussed in detail, whereas the highly similar data of duplexes **IV**_**X**_ are presented in the Supplementary Material (Fig. S3 and S4).

**Fig. 5:**
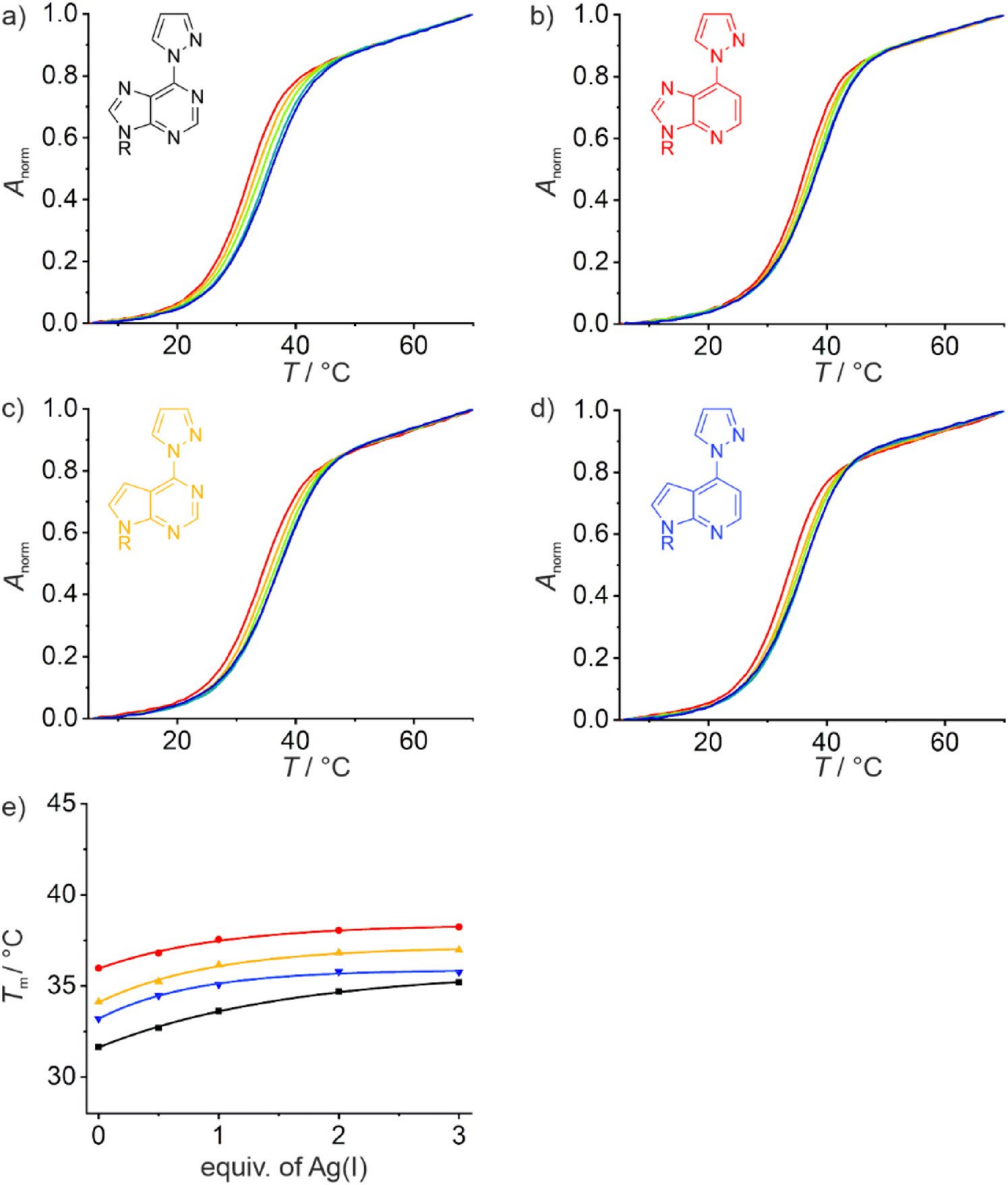
Melting curves of **a** duplex **III**_**6PP**_, **b** duplex ${\textbf{III}}_{{}^{{\textbf{1D}}}{\textbf{6PP}}}$, **c** duplex ${\textbf{III}}_{{}^{{\textbf{7D}}}{\textbf{6PP}}}$, and **d** duplex ${\textbf{III}}_{{}^{{\textbf{1,7D}}}{\textbf{6PP}}}$ with a thymine residue located opposite X. Color code: red, no Ag(I); yellow: 0.5 equiv. of Ag(I); green: 1 equiv. of Ag(I); turquoise: 2 equiv. of Ag(I); blue: 3 equiv. of Ag(I). **e**) Overview of the melting temperatures *T*_m_ of these duplexes depending on the amount of Ag(I). Chemical representations of the nucleobases X and the melting temperatures of their corresponding duplexes **I**_**X**_ are shown in the same color. For better comparison, Fig. 5e is drawn on the same scale as Fig. 3e. Conditions: 1 μM duplex, 5 mM MOPS (pH 6.8), 150 mM NaClO_4_

**Fig. 6:**
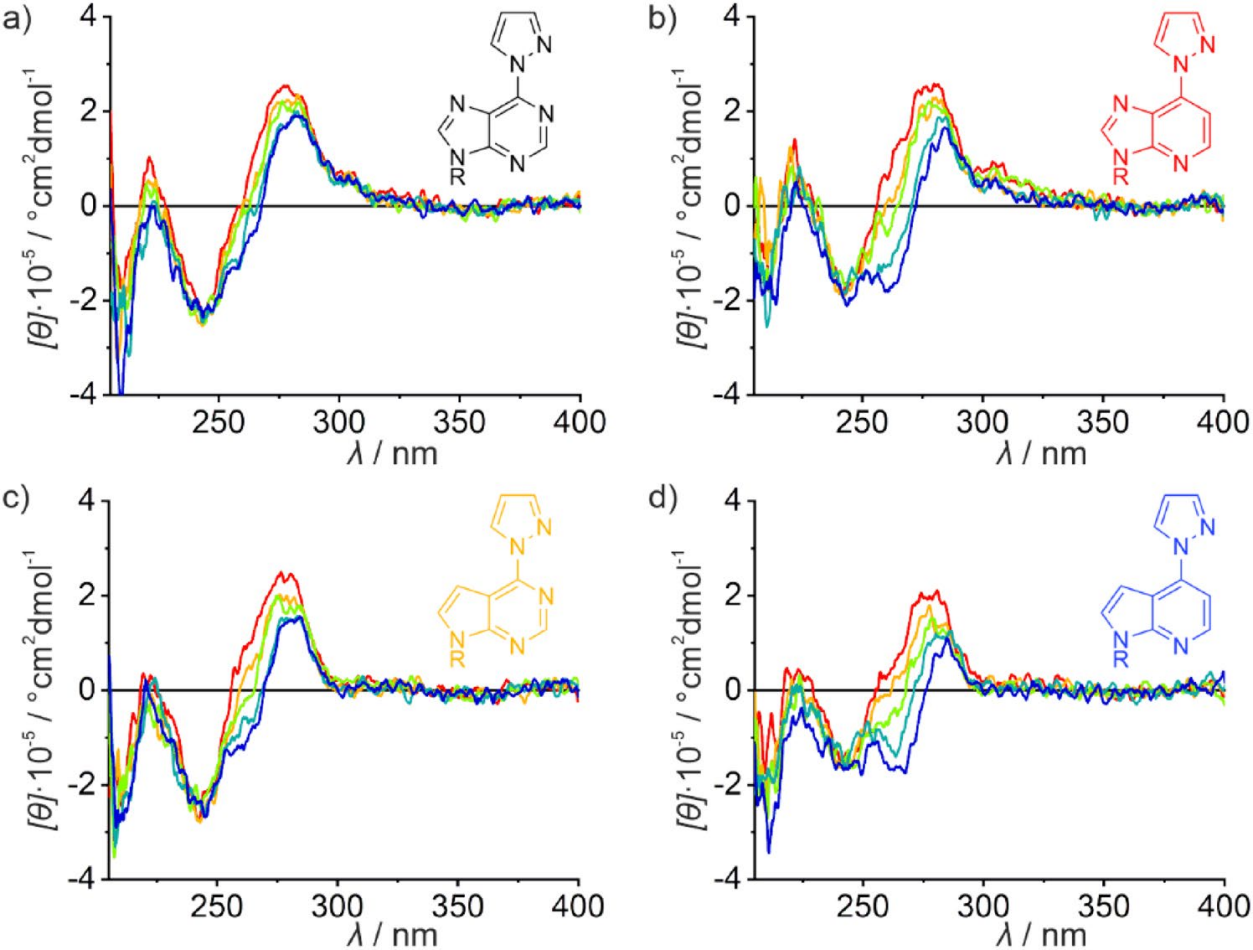
CD spectra of **a** duplex **III**_**6PP**_, **b** duplex ${\textbf{III}}_{{}^{{\textbf{1D}}}{\textbf{6PP}}}$, **c** duplex ${\textbf{III}}_{{}^{{\textbf{7D}}}{\textbf{6PP}}}$, and **d** duplex ${\textbf{III}}_{{}^{{\textbf{1,7D}}}{\textbf{6PP}}}$ with a thymine residue located opposite X in the presence of increasing amounts of Ag(I). Color code: red, no Ag(I); yellow: 0.5 equiv. of Ag(I); green: 1 equiv. of Ag(I); turquoise: 2 equiv. of Ag(I); blue: 3 equiv. of Ag(I). Conditions: 1 μM duplex, 5 mM MOPS (pH 6.8), 150 mM NaClO_4_, 5 ℃

An inspection of Fig. 5 and S3, showing the melting curves of duplexes **III**_X_ and **IV**_X_, respectively, in the presence of increasing amounts of Ag(I), clearly shows that the presence of Ag(I) does not significantly stabilize the duplexes. The plot of *T*_m_ *vs*. added equivalents of Ag(I) (Fig. 5e) rather shows a small asymptotic increase in *T*_m_. In the presence of one Ag(I) per duplex, Δ*T*_m_ amounts to merely 1–2 °C, rendering the selective formation of X–Ag(I)–T base pairs rather unlikely. In combination with the asymptotic increase of *T*_m_, these data strongly suggest a non-specific binding of the metal ion. This conclusion is further corroborated by the CD spectra (Fig. 6). Starting from a typical B-DNA-type CD spectrum, the decrease in intensity of the positive Cotton effect at ca. 280 nm accompanied by its red-shift is strongly suggesting the beginning of a structural rearrangement towards duplexes containing non-Watson–Crick-type Ag(I)-mediated base pairs involving canonical nucleobases [15, 56, 57]. Hence, an initial non-specific binding of Ag(I) can be concluded for duplexes **III**_X_ bearing a central X:T pair. An analogous behavior is observed for duplexes **IV**_X_ (Fig. S4), in which the central base pair is reversed with respect to duplexes **III**_X_.

As coordination of silver(I) by thymine requires prior deprotonation of the nucleobase (in contrast to coordination by cytosine) [36], duplexes **III**_**6PP**_ and **IV**_**6PP**_ were exemplarily investigated at pH 9.0, too (Fig. S5). In the absence of silver(I) ions, a minor (but not necessarily significant) decrease in melting temperature of 1–2 °C is observed in comparison to pH 6.8. This is quite expected, as an elevated pH is known to destabilize double-stranded DNA because of weakening hydrogen bonds [58]. Upon the addition of one Ag(I) per duplex, a stabilization Δ*T*_m_ of 5 °C is observed for **III**_**6PP**_ (3 °C for **IV**_**6PP**_). While this is certainly a larger increase in melting temperature than at pH 6.8, the asymptotic shape of the curve when plotting *T*_m_ vs. added equivalents of Ag(I) for duplex **III**_**6PP**_ (Fig. S5a) speaks out against a specific binding [42]. Surprisingly, even a decrease in *T*_m_ is observed for duplex **IV**_**6PP**_ once more than one Ag(I) per duplex is added (Fig. S5b). This decrease goes along with a very prominent change of the CD spectrum. As discussed above, this strongly suggests the onset of a structural rearrangement towards duplexes containing non-Watson–Crick-type Ag(I)-mediated base pairs of canonical nucleobases. It can be concluded that an increase in pH does not assist the formation of a highly stabilizing Ag(I)-mediated base pair between 6PP and thymine. We therefore refrained from extending the characterization of the Ag(I)-binding properties under alkaline conditions to the deaza derivatives of 6PP.

### Comparison of the melting temperatures

To allow an easy comparison of the melting temperatures *T*_m_ and their dependence on Ag(I), Table 1 presents an overview of all relevant melting temperatures determined in this study. As discussed above, only 6PP and ^7D^6PP are able to form significantly stabilizing Ag(I)-mediated base pairs with cytosine. In general, the resulting X–Ag(I)–C base pair appears to be slightly more stabilizing when the purine derivative X is located in the pyrimidine-rich strand of the duplex (i.e. duplexes **I**_X_) rather than in the purine-rich strand (i.e. duplexes **II**_X_). A sequence-dependence of the stability of a metal-mediated base pair is not unprecedented [59]. It is in good agreement with the findings of a previous study on Ag(I)-mediated X–Ag(I)–Y base pairing, where X and Y are 6PP, ^1D^6PP, ^7D^6PP, or ^1,7D^6PP [43]. That study also shows a particularly high stability of Ag(I)-mediated base pairs involving 6PP or ^7D^6PP in the pyrimidine-rich strand of the duplex.

**Table 1:** Duplex melting temperatures *T*_m_/℃ in the absence and presence of Ag(I) and change in melting temperature Δ*T*_m_/℃ upon the addition of one equivalent of Ag(I)^a^

Duplex	*T*_m_/℃ (no Ag(I))	*T*_m_/℃ (1 equiv. of Ag(I))	Δ*T*_m_/℃ (0 → 1 equiv. of Ag(I))
**I**_**6PP**_	30	41	11
${\textbf{I}}_{{}^{{\textbf{1D}}}{\textbf{6PP}}}$	29	32	3
${\textbf{I}}_{{}^{{\textbf{7D}}}{\textbf{6PP}}}$	31	39	8
${\textbf{I}}_{{}^{{\textbf{1,7D}}}{\textbf{6PP}}}$	29	32	3
**II**_**6PP**_	29	38	9
${\textbf{II}}_{{}^{{\textbf{1D}}}{\textbf{6PP}}}$	30	32	2
${\textbf{II}}_{{}^{{\textbf{7D}}}{\textbf{6PP}}}$	30	36	6
${\textbf{II}}_{{}^{{\textbf{1,7D}}}{\textbf{6PP}}}$	29	32	3
**III**_**6PP**_	32/31^b^	34/36^b^	2/5^b^
${\textbf{III}}_{{}^{{\textbf{1D}}}{\textbf{6PP}}}$	37	38	1
${\textbf{III}}_{{}^{{\textbf{7D}}}{\textbf{6PP}}}$	34	36	2
${\textbf{III}}_{{}^{{\textbf{1,7D}}}{\textbf{6PP}}}$	33	35	2
**IV**_**6PP**_	30/28^b^	32/31^b^	2/3^b^
${\textbf{IV}}_{^{\textbf{1D}}{\textbf{6PP}}}$	33	35	2
${\textbf{IV}}_{^{\textbf{7D}}{\textbf{6PP}}}$	31	33	2
${\textbf{IV}}_{{}^{{\textbf{1,7D}}}{\textbf{6PP}}}$	30	33	3

^a^Conditions: 1 μM duplex, 5 mM MOPS (pH 6.8), 150 mM NaClO_4_; estimated standard error of *T*_m_: 1 ℃

^b^Experimental conditions for the second value: 1 μM duplex, 5 mM borate (pH 9.0), 150 mM NaClO_4_; estimated standard error of *T*_m_: 1 ℃

### Comparison of the CD spectra

In the absence of Ag(I), the CD spectra of all duplexes under investigation resemble that of a typical B-type DNA duplex. Upon increasing amounts of Ag(I), a red-shift of the positive Cotton effect at ca. 280 nm is observed for most duplexes, accompanied by a decreasing molar ellipticity. For some duplexes, a new negative Cotton effect starts to emerge at ca. 260 nm. As stated above, such spectroscopic features were previously observed for duplexes composed of Ag(I)-mediated non-Watson–Crick base pairs with canonical nucleobases [15, 56, 57]. Considering that duplexes **I** – **IV** are essentially identical (except for their sequence direction and the identity of their central artificial base pair), one would expect that an additional non-specific binding should be the same for all duplexes and merely start at higher Ag(I) concentrations for those duplexes containing a high-affinity Ag(I)-binding site. Fascinatingly, duplexes **I**_**6PP**_ and ${\textbf{I}}_{{}^{{\textbf{7D}}}{\textbf{6PP}}}$ do not show any of these changes at all (Fig. 4a, c), and they are less pronounced for duplex **II**_**6PP**_. As a first approximation, those duplexes that are most stabilized by the Ag(I)-mediated base pair show the least propensity to transition to a silver(I)-containing non-Watson–Crick duplex structure in the presence of excess Ag(I). Accordingly, duplex ${\textbf{III}}_{{}^{{\textbf{1D}}}{\textbf{6PP}}}$, which is the least Ag(I)-stabilized duplex in the reported set of experiments, is amongst those with the most prominent CD-spectroscopic changes (Fig. 6b). This rule of thumb is in good agreement with earlier reported data on silver(I)-mediated homo base pair formation of 6PP and its deaza derivatives [42]. In that study, a lack of CD-spectroscopic changes in the presence of excess Ag(I) was observed only for those duplexes that form stable Ag(I)-mediated base pairs. Likewise, elevated pH values typically destabilize DNA duplexes. Accordingly, the CD-spectroscopic changes are even more prominent at pH 9.0 compared to pH 6.8 (cf. Figure 6a and S5a; S4a and S5b). This could mean that excess silver(I) ions do *not* bind non-specifically to the canonical nucleobases (e.g. the N7 positions of the purine residues), but that some binding specificity exists, also depending on the total duplex stability (and probably additional as yet undetermined factors). Further investigations, which are beyond the scope of the present study, are ongoing to unravel the fascinating transition of canonical B-DNA duplexes to a silver(I)-containing non-Watson–Crick duplex in the presence of excess Ag(I).

### Proposed base pairing pattern

**Fig. 7:**
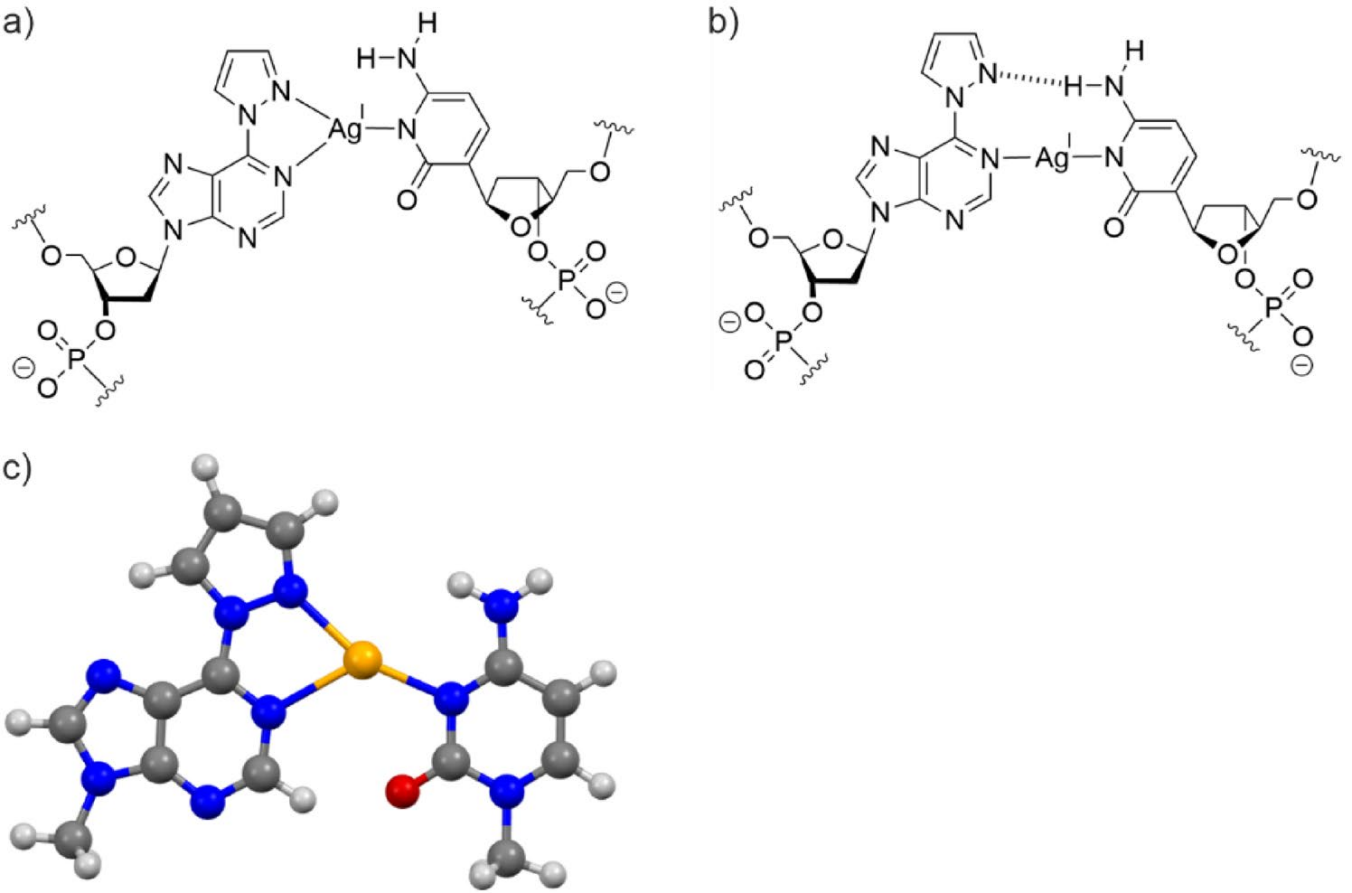
Possible structures of the 6PP–Ag(I)–C base pair. It was initially considered that the base pair could contain **a** an Ag(I) ion in a trigonal coordination environment or **b** a linearly coordinated Ag(I) ion and a synergistic hydrogen bond. **c** According to DFT calculations, the structure with a trigonally coordinated Ag(I) ion is the lowest-energy one

DFT calculations were performed to determine the most likely structures of the 6PP–Ag(I)–C and ^7D^6PP–Ag(I)–C base pairs. In principle, two structures appeared to be reasonable. As it can be assumed that the bonding pattern is identical for 6PP–Ag(I)–C and ^7D^6PP–Ag(I)–C, the two initially considered structures are depicted in Fig. 7 for 6PP–Ag(I)–C only. These structures were used as starting points for the DFT calculations.

The calculations showed that the proposed structure involving a synergistic hydrogen bond is less stable. Irrespective of which starting geometry is used, the calculations converge to the structure shown in Fig. 7c for the 6PP–Ag(I)–C pair. The same observation is made for the ^7D^6PP–Ag(I)–C base pair, which also adopts this type of geometry (see Fig. S6 for all calculated structures). Interestingly, the calculations show that the formation of a 6PP–Ag(I)–C pair from the silver(I)-free mispair is more stabilizing by ca. 5.5 kJ mol^–1^ than the formation of the ^7D^6PP–Ag(I)–C pair. This is in good agreement with the experimentally observed larger thermal stabilization of the former base pair (ΔΔ*T*_m_ = 3 ℃).

As metal-mediated base pairs can principally also include hydrogen-bonded water molecules [60, 61], we also investigated the possibility of the presence of one or two explicit water molecules in the 6PP–Ag(I)–C pair. However, energetically relevant water-containing metal-mediated base pairs turned out to be significantly non-planar (Fig. S7), suggesting that they are not formed within the oligonucleotide duplexes.

## Conclusion

Out of the eight possible base pairs under investigation (X:C and X:T, with X = 6PP, ^1D^6PP, ^7D^6PP, or ^1,7D^6PP), only 6PP:C and ^7D^6PP:C were found to form stable Ag(I)-mediated base pairs. Duplexes containing any of the other combinations were not significantly stabilized in the presence of equimolar amounts of Ag(I) ions.

With respect to the initially proposed tentative application of 6PP and its derivatives in the differentiation of the canonical pyrimidine nucleobases, two conclusions can be drawn. First, 6PP and ^7D^6PP are indeed able to distinguish cytosine from thymine via silver(I)-mediated base pairing. However, they are outcompeted by the previously reported artificial nucleobase 1*H*-imidazo-[4,5-*f*][1,10]phenanthroline [36, 37], which also forms stabilizing Ag(I)-mediated base pairs with cytosine but in addition leads to a duplex destabilization when paired with thymine in the presence of Ag(I). As in the case of the phenanthroline derivative a simplified DNA backbone was used for the artificial nucleoside, it will be an interesting subject of future studies to evaluate whether its superior ability to discriminate between the pyrimidine nucleosides is a result of the different ligand or of the different backbone with respect to 6PP and ^7D^6PP. Nevertheless, the simple and straightforward synthetic access to 6PP still makes this nucleobase a potential candidate for the detection of single-nucleotide polymorphisms (SNPs). Second, the additional bulk present in the previously reported 3,5-dimethylpyrazole-substituted purines [40] is not required for a differentiation of cytosine and thymine. Again, the synthetic ease is certainly an advantage of 6PP.

In summary, two artificial purine-derived nucleobases have been successfully applied in the discrimination of cytosine and thymine via the formation of Ag(I)-mediated base pairs. This feature might find an application in the selective detection of oligonucleotide sequences.

### Supplementary Information

Below is the link to the electronic supplementary material.

Supplementary file1 (PDF 2169 KB)

## Data Availability

The data that support the findings of this study are available from the corresponding author upon reasonable request.
