# Supplementary material for: 6-Pyrazolylpurine and its deaza derivatives as nucleobases for silver(I)-mediated base pairing with pyrimidines
Source: J Biol Inorg Chem. 2023 Nov 20;28(8):791–803. doi: 10.1007/s00775-023-02022-0 (PMC10687122; doi:10.1007/s00775-023-02022-0)
Supplement: Supplementary file 1 — Supplementary file1 (PDF 2169 KB) [file 775_2023_2022_MOESM1_ESM.pdf]

Supplementary Material

6-Pyrazolylpurine and its Deaza Derivatives as Nucleobases for Silver(I)-Mediated Base Pairing with Pyrimidines

Daniela Escher,[a] Tim Schäfer,[a] Marian Hebenbrock[a] and Jens Müller*[a,b]

[a] Dr. D. Escher, T. Schäfer, Dr. M. Hebenbrock, Prof. Dr. J. Müller

Universität Münster

Institut für Anorganische und Analytische Chemie

Corrensstr. 30, 48149 Münster (Germany)

E-mail: mueller.j@uni-muenster.de

[b] Prof. Dr. J. Müller

Universität Münster

Center for Soft Nanoscience (SoN) and Cells in Motion Interfaculty Centre (CiMIC)

Corrensstr. 30, 48149 Münster (Germany)

Table of contents

Fig. S1 Melting curves of duplexes **II$_{6PP}$**, **II$_{1D6PP}$**, **II$_{7D6PP}$**, and **II$_{1,7D6PP}$** S2

Fig. S2 CD spectra of duplexes **II$_{6PP}$**, **II$_{1D6PP}$**, **II$_{7D6PP}$**, and **II$_{1,7D6PP}$** S3

Fig. S3 Melting curves of duplexes **IV$_{6PP}$**, **IV$_{1D6PP}$**, **IV$_{7D6PP}$**, and **IV$_{1,7D6PP}$** S4

Fig. S4 CD spectra of duplexes **IV$_{6PP}$**, **IV$_{1D6PP}$**, **IV$_{7D6PP}$**, and **IV$_{1,7D6PP}$** S5

Fig. S5 Melting curves and CD spectra of duplexes **III$_{6PP}$** and **IV$_{6PP}$** at pH 9.0 .... S6

Fig. S6 DFT-optimized geometries of 6PP:C, ^{7D}6PP:C, 6PP–Ag(I)–C, and ^{7D}6PP–Ag(I)–C S7

Fig. S7 DFT-optimized geometries of 6PP–Ag(I)–C with explicit water S8

Table S1 Mass-spectrometric characterization of the oligonucleotides S9

Fig. S8 Mass spectrum of ODN1 .... S10

Fig. S9 Mass spectrum of ODN2 .... S10

Fig. S10 Mass spectrum of ODN3 .... S11

Fig. S11 Mass spectrum of ODN4 .... S11

Fig. S12 Mass spectrum of ODN5 .... S12

Fig. S13 Mass spectrum of ODN6 .... S12

Fig. S14 Mass spectrum of ODN7 .... S13

Fig. S15 Mass spectrum of ODN8 .... S13

Fig. S16 Mass spectrum of ODN9 .... S14

Fig. S17 Mass spectrum of ODN10 .... S14

Fig. S18 Mass spectrum of ODN11 .... S15

Fig. S19 Mass spectrum of ODN12 .... S15

xyz coordinates of 6PP:C S16

xyz coordinates of ^{7D}6PP:C S18

xyz coordinates of 6PP–Ag(I)–C S20

xyz coordinates of ^{7D}6PP–Ag(I)–C S22

xyz coordinates of 6PP–Ag(I)–C × H_2O (structure **I**) S24

xyz coordinates of 6PP–Ag(I)–C × H_2O (structure **II**) S26

xyz coordinates of 6PP–Ag(I)–C × H_2O (structure **III**) S28

xyz coordinates of 6PP–Ag(I)–C × 2 H_2O (structure **IV**) S30

xyz coordinates of 6PP–Ag(I)–C × 2 H_2O (structure **V**) S32

xyz coordinates of H_2O S34

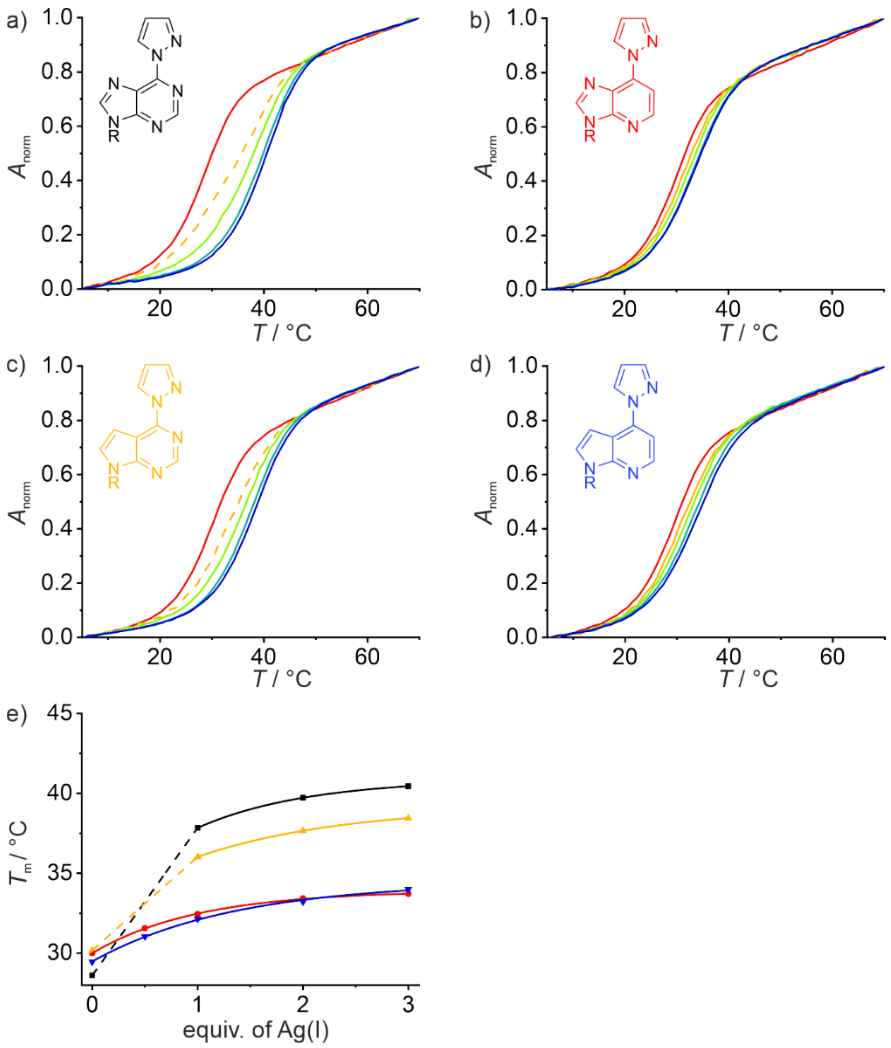

Fig. S1 Melting curves of a) duplex $\mathbf{II}_{\mathbf{6PP}}$, b) duplex $\mathbf{II}_{\mathbf{1D6PP}}$, c) duplex $\mathbf{II}_{\mathbf{7D6PP}}$, and d) duplex $\mathbf{II}_{\mathbf{1,7D6PP}}$. Color code: red, no Ag(I); yellow: 0.5 equiv. of Ag(I); green: 1 equiv. of Ag(I); turquoise: 2 equiv. of Ag(I); blue: 3 equiv. of Ag(I). A melting curve plotted as a broken line indicates biphasic melting behavior. e) Overview of the melting temperatures T_m of these duplexes depending on the amount of Ag(I). Broken lines indicate biphasic melting, hence no melting temperature can be determined in this region. Chemical representations of the nucleobases X and the melting temperatures of their corresponding duplexes $\mathbf{II}_{\mathbf{X}}$ are shown in the same color. For better comparison, Figure S1e is drawn on the same scale as Figure 3e. Conditions: 1 µM duplex, 5 mM MOPS (pH 6.8), 150 mM $NaClO_4$.

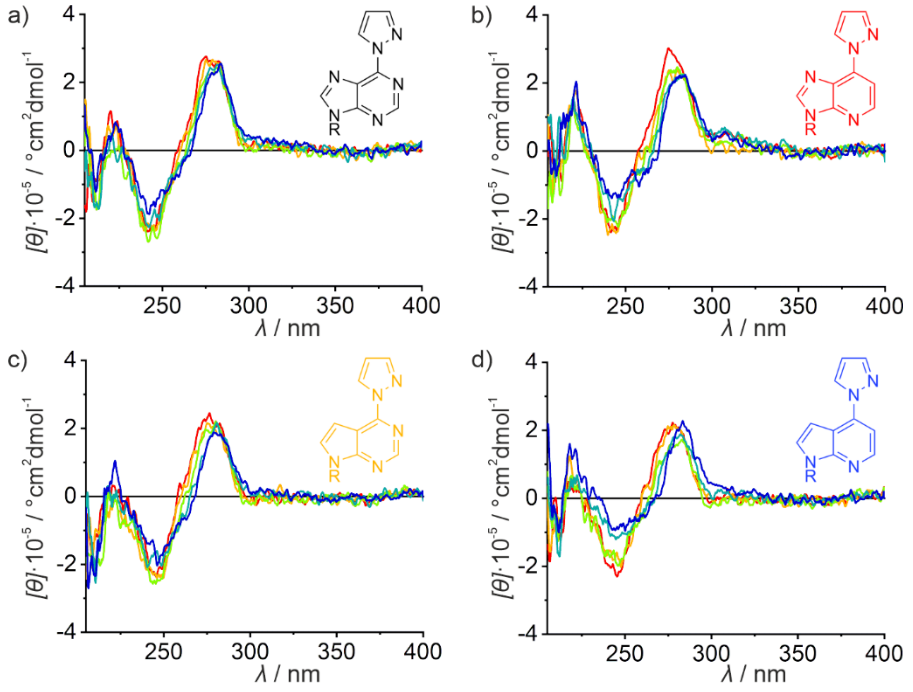

Fig. S2 CD spectra of a) duplex **II**$_{\mathbf{6PP}}$, b) duplex **II**$_{\mathbf{1D6PP}}$, c) duplex **II**$_{\mathbf{7D6PP}}$, and d) duplex **II**$_{\mathbf{1,7D6PP}}$ in the presence of increasing amounts of Ag(I). Color code: red, no Ag(I); yellow: 0.5 equiv. of Ag(I); green: 1 equiv. of Ag(I); turquoise: 2 equiv. of Ag(I); blue: 3 equiv. of Ag(I). Conditions: 1 μM duplex, 5 mM MOPS (pH 6.8), 150 mM $NaClO_4$, 5 °C.

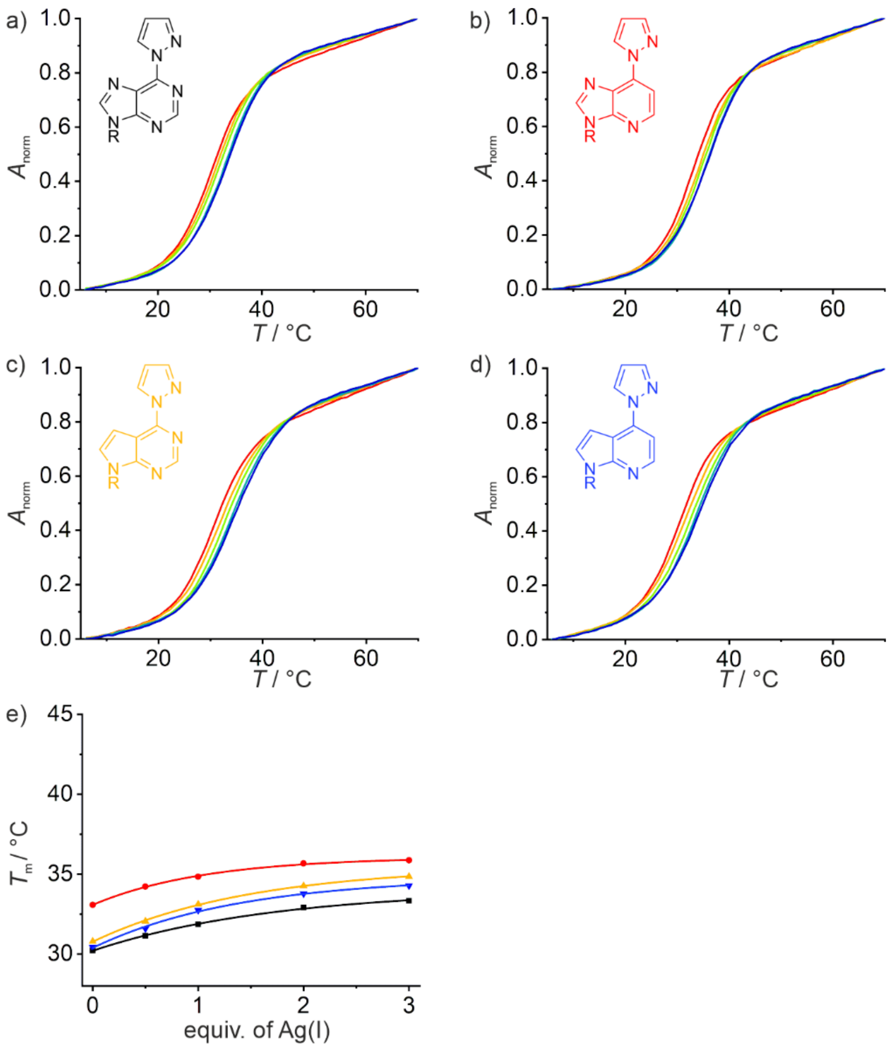

Fig. S3 Melting curves of a) duplex **IV**$_{6PP}$, b) duplex **IV**$_{1D6PP}$, c) duplex **IV**$_{7D6PP}$, and d) duplex **IV**$_{1,7D6PP}$. Color code: red, no Ag(I); yellow: 0.5 equiv. of Ag(I); green: 1 equiv. of Ag(I); turquoise: 2 equiv. of Ag(I); blue: 3 equiv. of Ag(I). e) Overview of the melting temperatures T_m of these duplexes depending on the amount of Ag(I). Chemical representations of the nucleobases X and the melting temperatures of their corresponding duplexes **IV**$_X$ are shown in the same color. For better comparison, Figure S3e is drawn on the same scale as Figure 3e. Conditions: 1 µM duplex, 5 mM MOPS (pH 6.8), 150 mM $NaClO_4$.

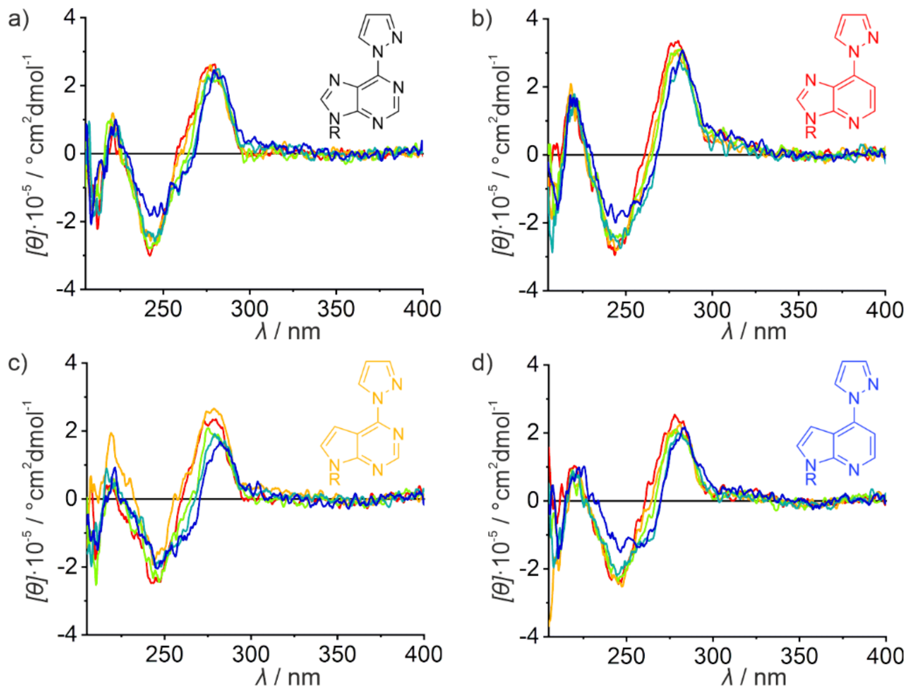

Fig. S4 CD spectra of a) duplex **IV**$_{\mathbf{6PP}}$, b) duplex **IV**$_{\mathbf{1D6PP}}$, c) duplex **IV**$_{\mathbf{7D6PP}}$, and d) duplex **IV**$_{\mathbf{1,7D6PP}}$ in the presence of increasing amounts of Ag(I). Color code: red, no Ag(I); yellow: 0.5 equiv. of Ag(I); green: 1 equiv. of Ag(I); turquoise: 2 equiv. of Ag(I); blue: 3 equiv. of Ag(I). Conditions: 1 µM duplex, 5 mM MOPS (pH 6.8), 150 mM $NaClO_4$, 5 °C.

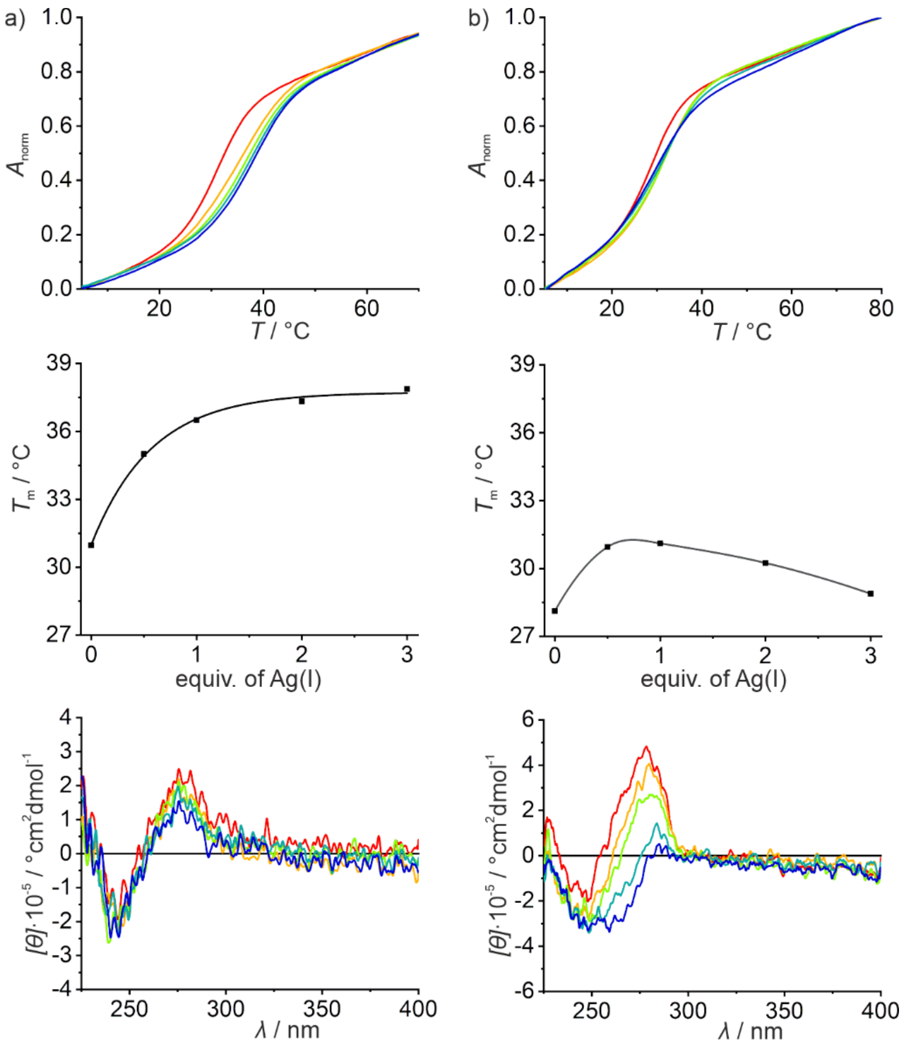

Fig. S5 Melting curves (top), melting temperature T_m (middle) and CD spectra (bottom) of a) duplex **III$_{6PP}$** and b) duplex **IV$_{6PP}$** in the presence of increasing amounts of Ag(I). Color code: red, no Ag(I); yellow: 0.5 equiv. of Ag(I); green: 1 equiv. of Ag(I); turquoise: 2 equiv. of Ag(I); blue: 3 equiv. of Ag(I). Conditions: 1 μM duplex, 5 mM borate (pH 9.0), 150 mM $NaClO_4$. CD spectra were recorded at 5 °C.

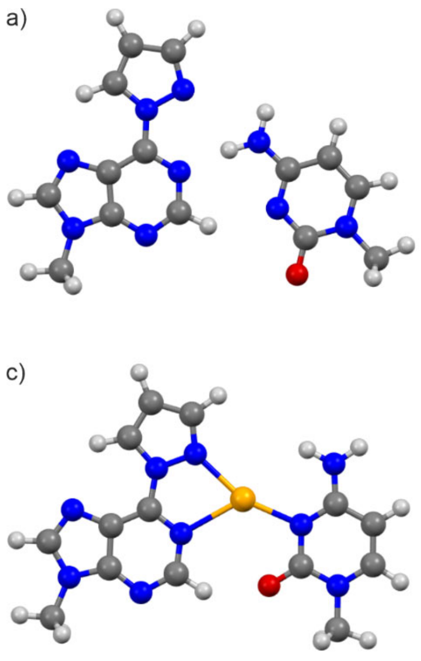

Figure S6 DFT-optimized geometries of a) 6PP:C, b) ^{7D}6PP:C, c) 6PP–Ag(I)–C, d) ^{7D}6PP–Ag(I)–C.

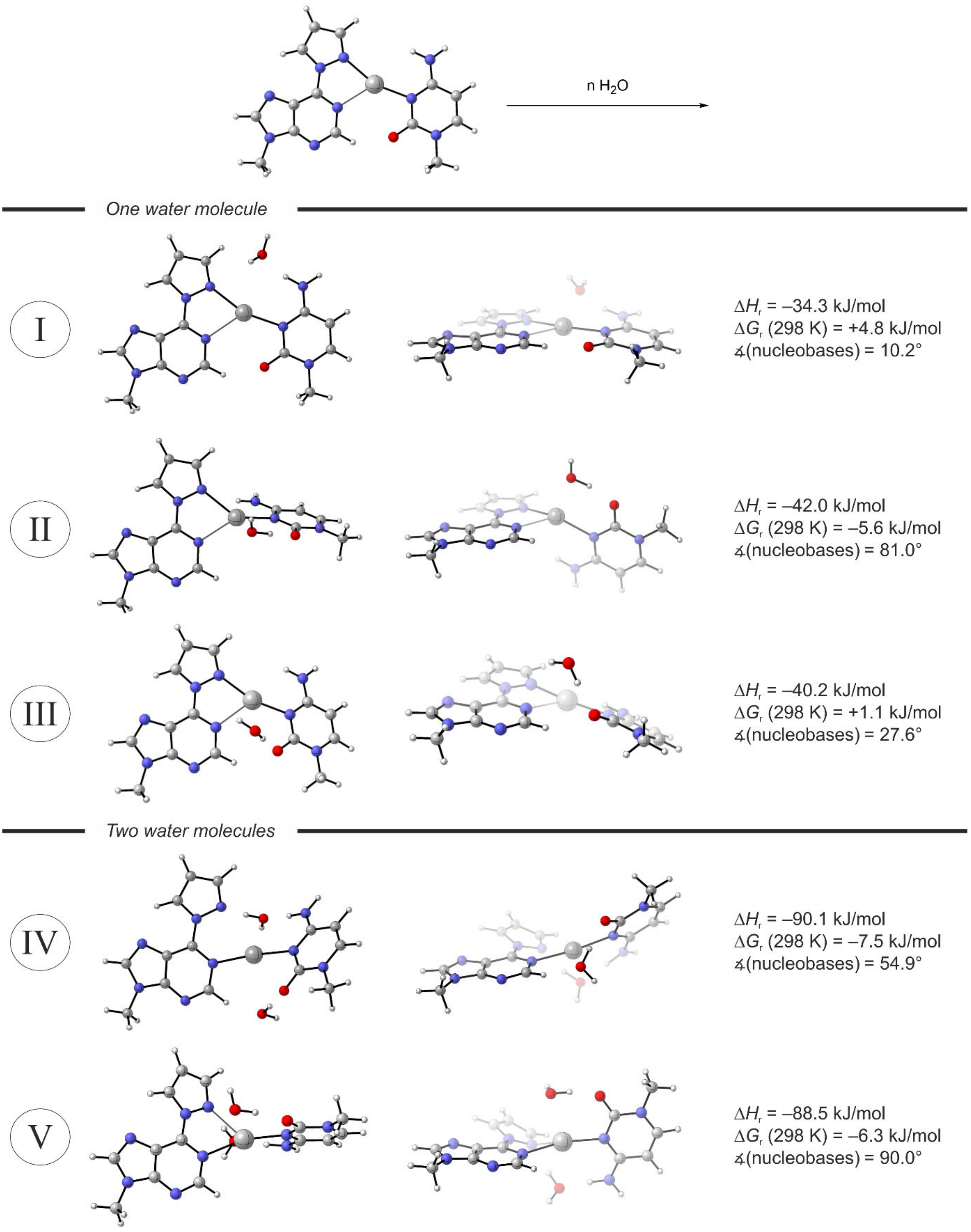

Figure S7 DFT-optimized geometries the 6PP–Ag(I)–C base pair containing one or two explicit water molecules, including their respective ΔH_r and ΔG_r as well as the angle between the two nucleobases. Different local minimum structures are depicted.

Tabe S1 Mass-spectrometric characterization of the oligonucleotides

Number	Sequence	Composition	$[M+H]^+$ (calcd.)	$[M+H]^+$ (found)	Figure
ODN1	5'-d(GAG GGA **6PP**AG AAA G)	$C_{133}H_{159}N_{66}O_{69}P_{12}$	4157	4157	S6
ODN2	5'-d(GAG GGA ^{7D}**6PP**AG AAA G)	$C_{134}H_{160}N_{65}O_{69}P_{12}$	4156	4156	S7
ODN3	5'-d(GAG GGA ^{1D}**6PP**AG AAA G)	$C_{134}H_{160}N_{65}O_{69}P_{12}$	4156	4155	S8
ODN4	5'-d(GAG GGA ^{1,7D}**6PP**AG AAA G)	$C_{135}H_{161}N_{64}O_{69}P_{12}$	4155	4155	S9
ODN5	5'-d(CTT TCT **6PP**TC CCT C)	$C_{127}H_{165}N_{36}O_{81}P_{12}$	3863	3863	S10
ODN6	5'-d(CTT TCT ^{7D}**6PP**TC CCT C)	$C_{128}H_{166}N_{35}O_{81}P_{12}$	3862	3862	S11
ODN7	5'-d(CTT TCT ^{1D}**6PP**TC CCT C)	$C_{128}H_{166}N_{35}O_{81}P_{12}$	3862	3862	S12
ODN8	5'-d(CTT TCT ^{1,7D}**6PP**TC CCT C)	$C_{129}H_{167}N_{34}O_{81}P_{12}$	3861	3862	S13
ODN9	5'-d(CTT TCT **C**TC CCT C)	$C_{123}H_{164}N_{33}O_{82}P_{12}$	3788	3788	S14
ODN10	5'-d(CTT TCT **T**TC CCT C)	$C_{124}H_{165}N_{32}O_{83}P_{12}$	3803	3804	S15
ODN11	5'-d(GAG GGA **C**AG AAA G)	$C_{129}H_{158}N_{63}O_{70}P_{12}$	4082	4081	S16
ODN12	5'-d(GAG GGA **T**AG AAA G)	$C_{130}H_{159}N_{62}O_{71}P_{12}$	4097	4097	S17

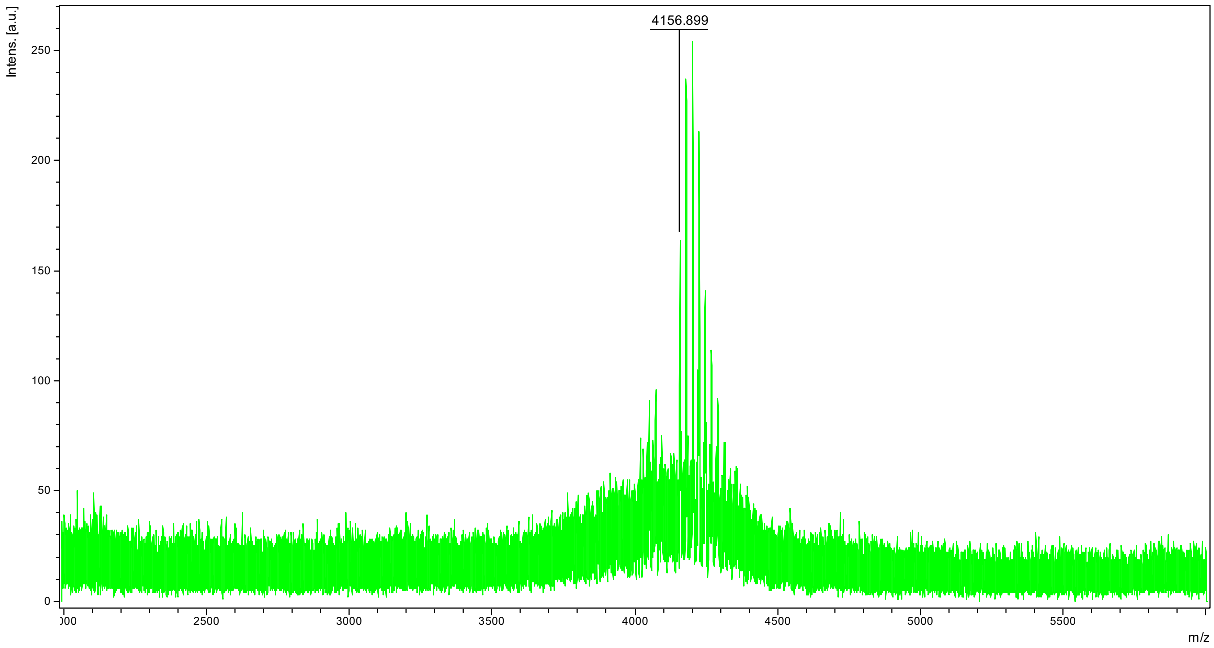

Fig. S8 Mass spectrum of ODN1.

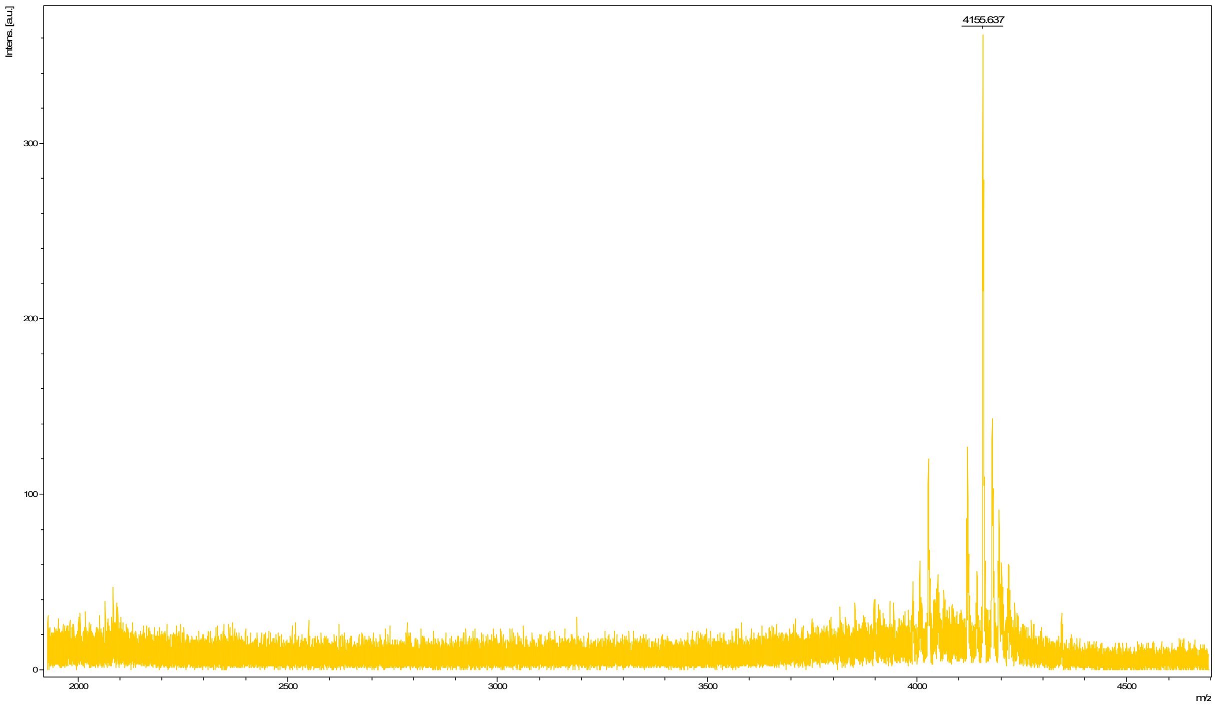

Fig. S9 Mass spectrum of ODN2.

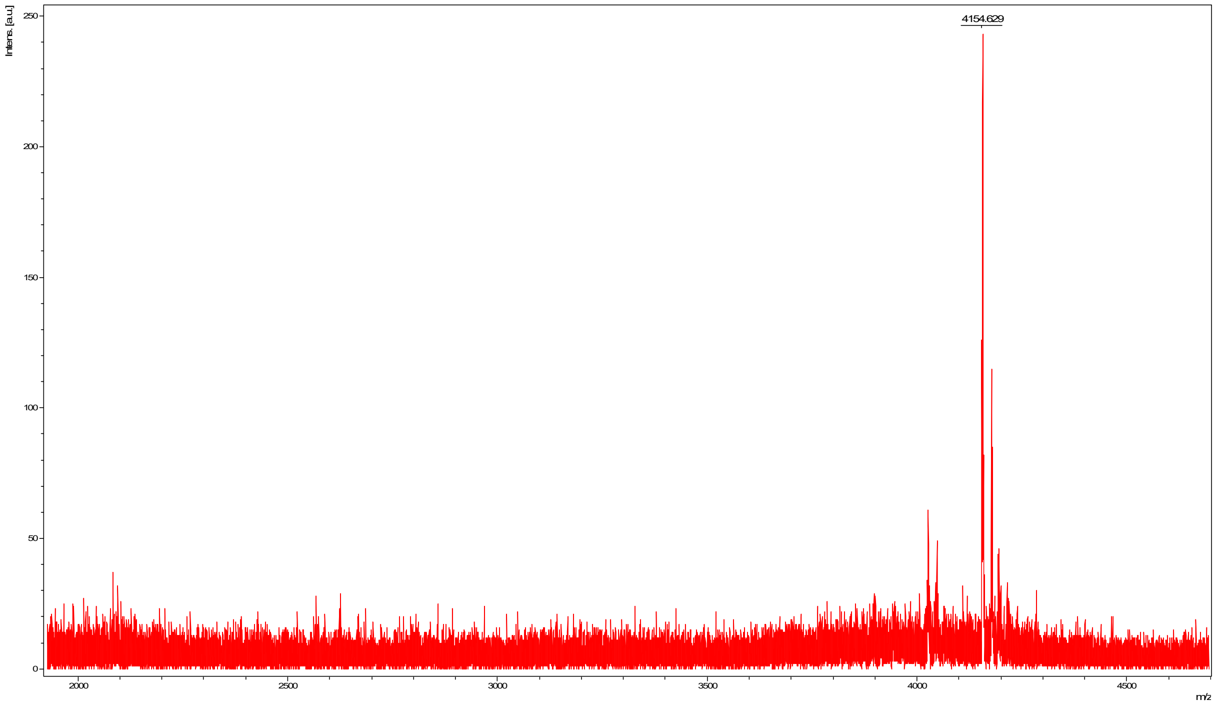

Fig. 10 Mass spectrum of ODN3.

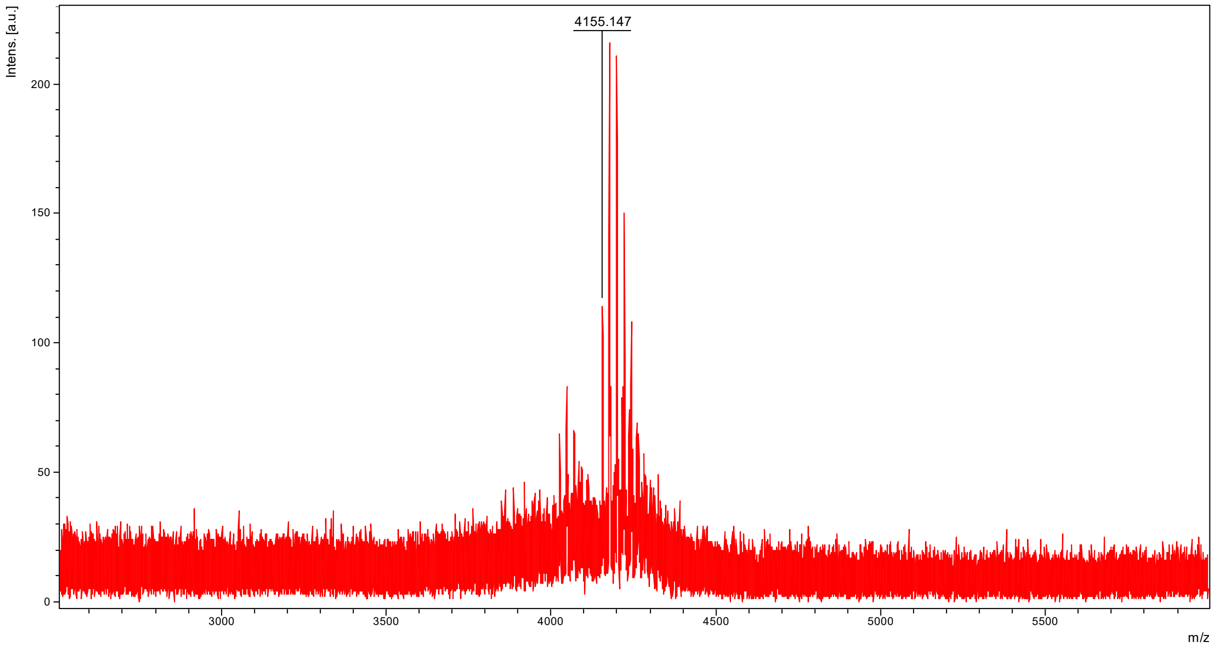

Fig. S11 Mass spectrum of ODN4.

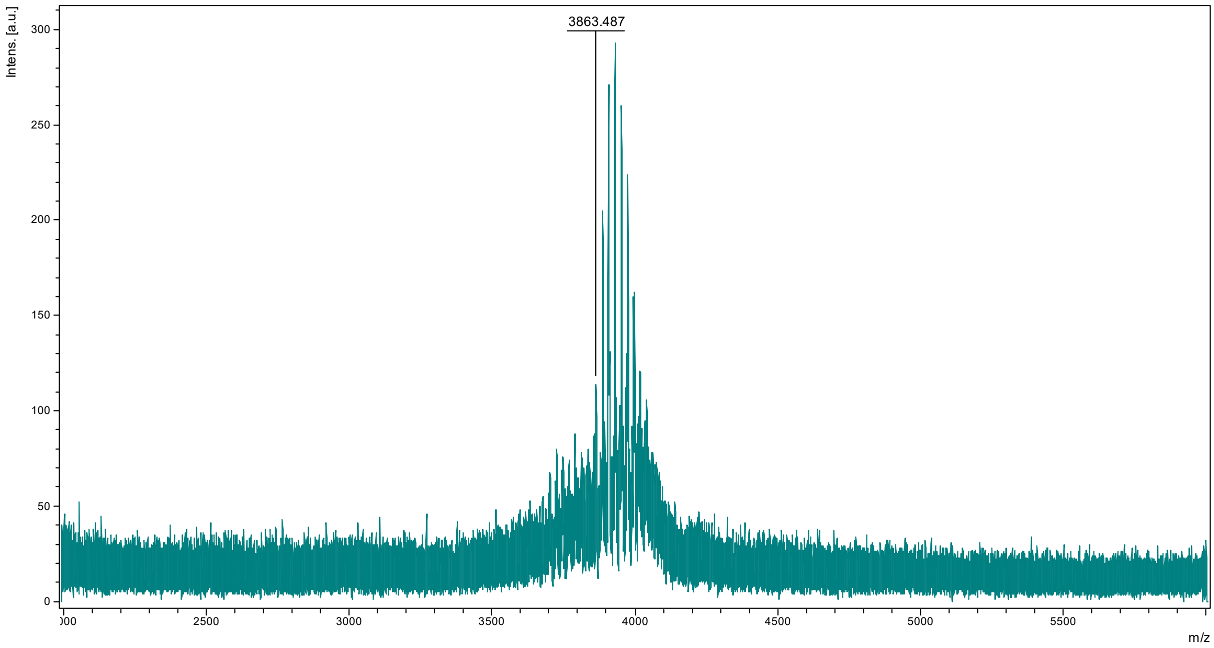

Fig. S12 Mass spectrum of ODN5.

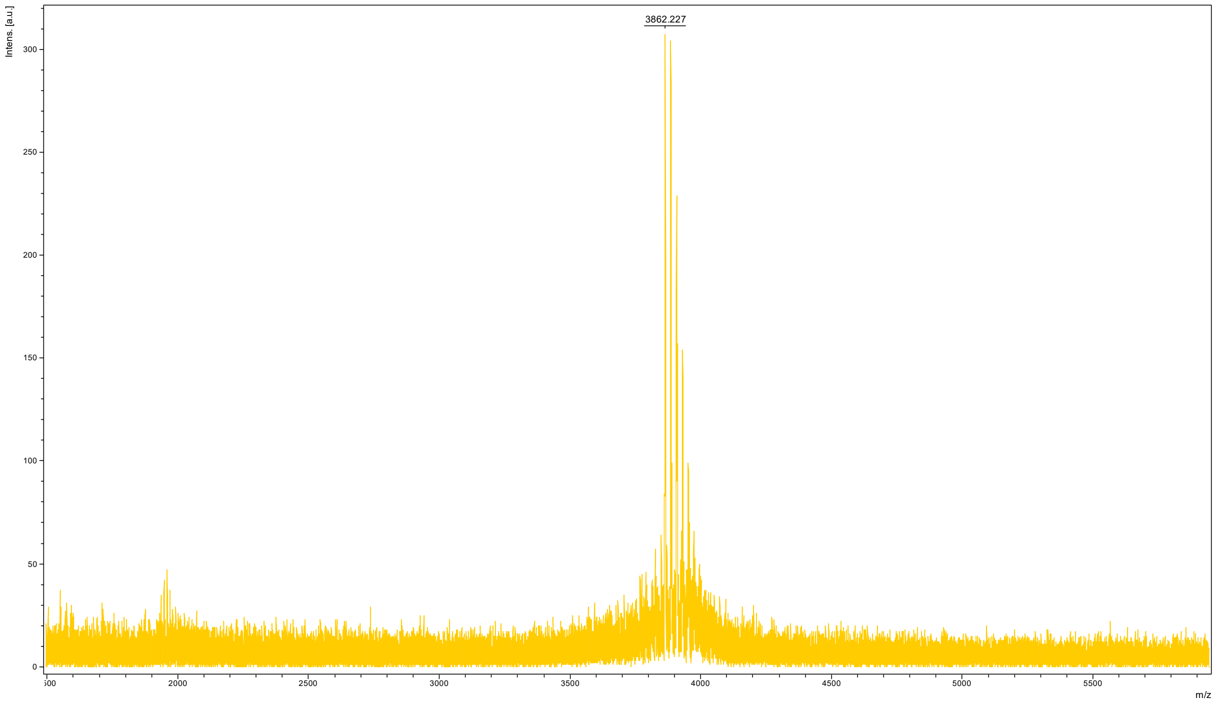

Fig. S13 Mass spectrum of ODN6.

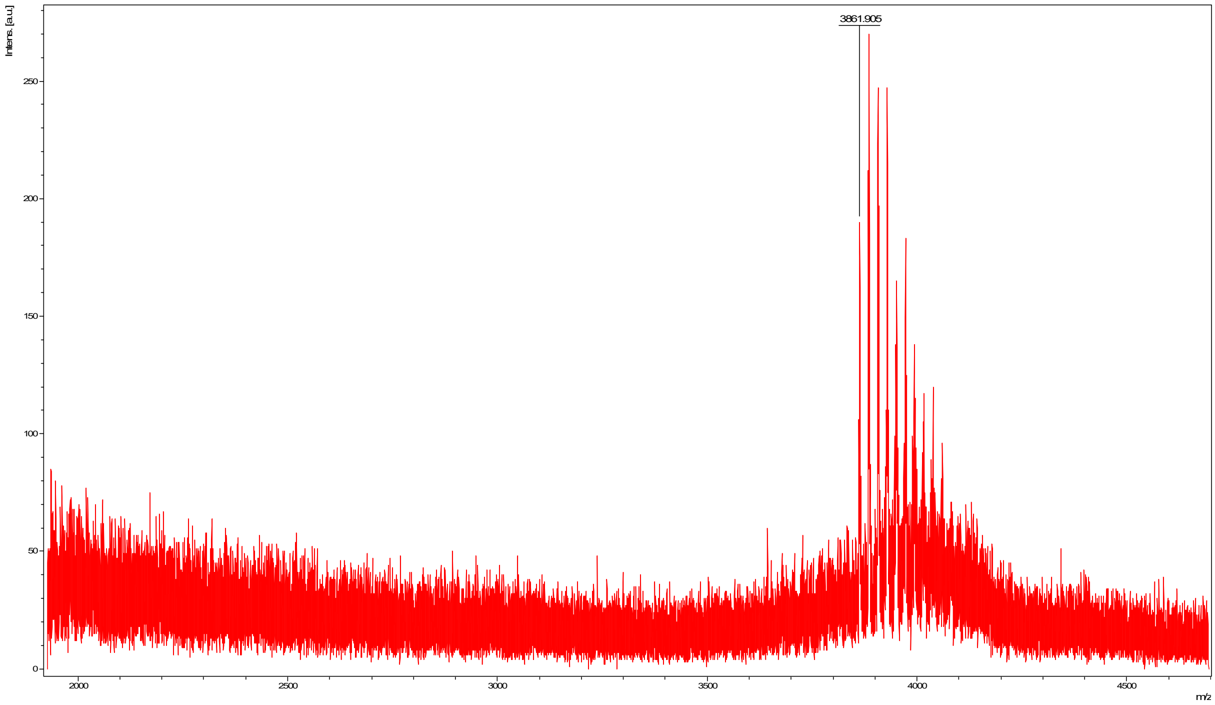

Fig. S14 Mass spectrum of ODN7.

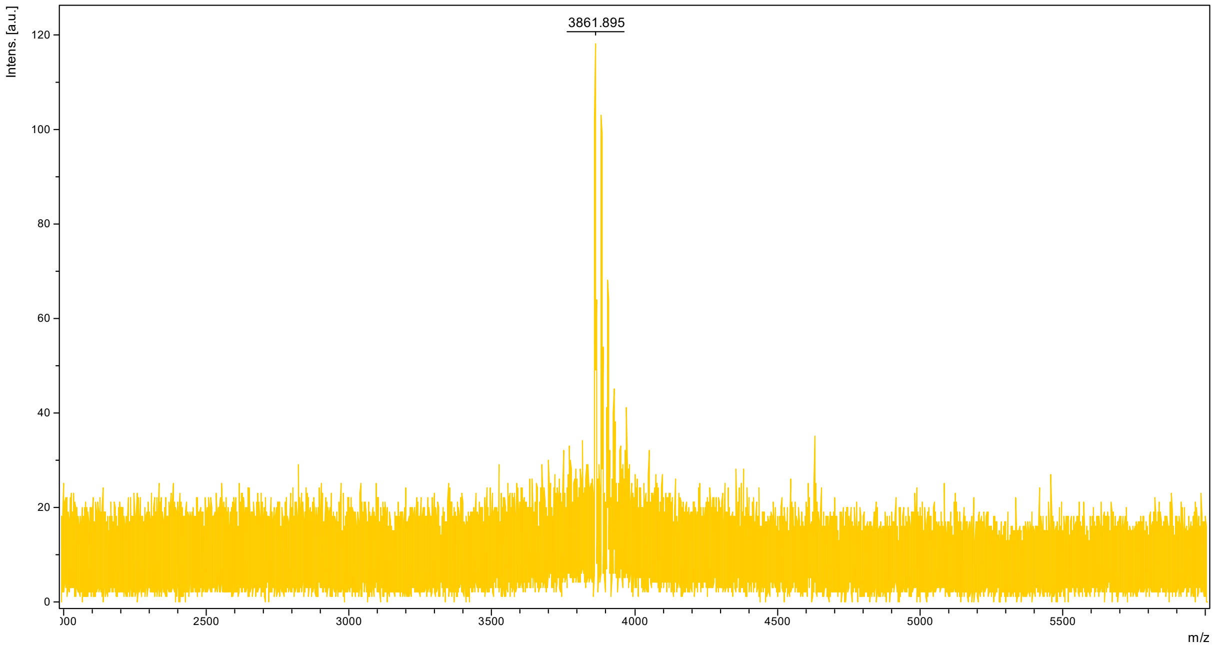

Fig. S15 Mass spectrum of ODN8.

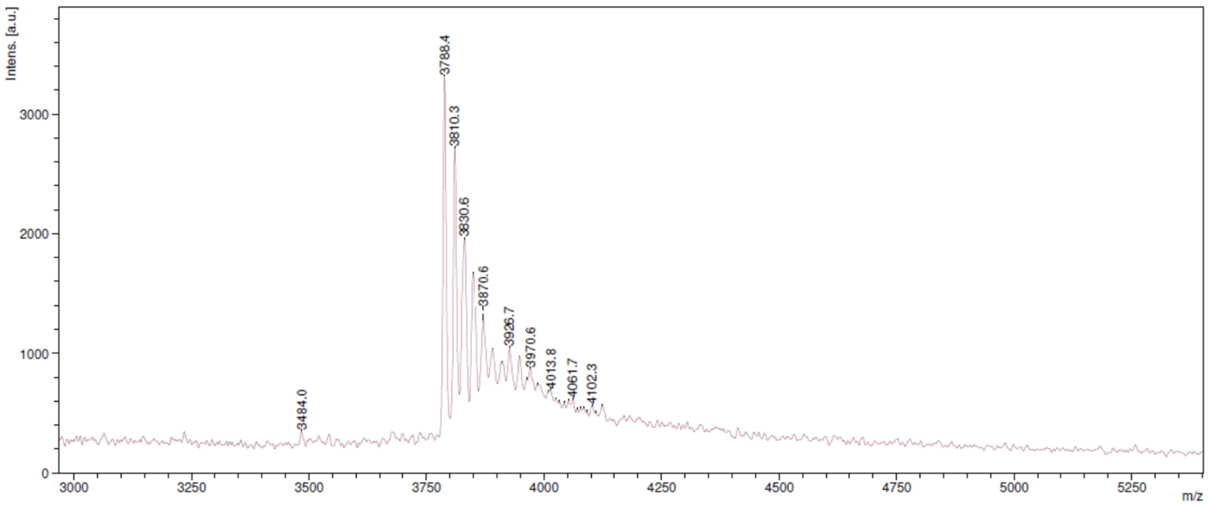

Fig. S16 Mass spectrum of ODN9.

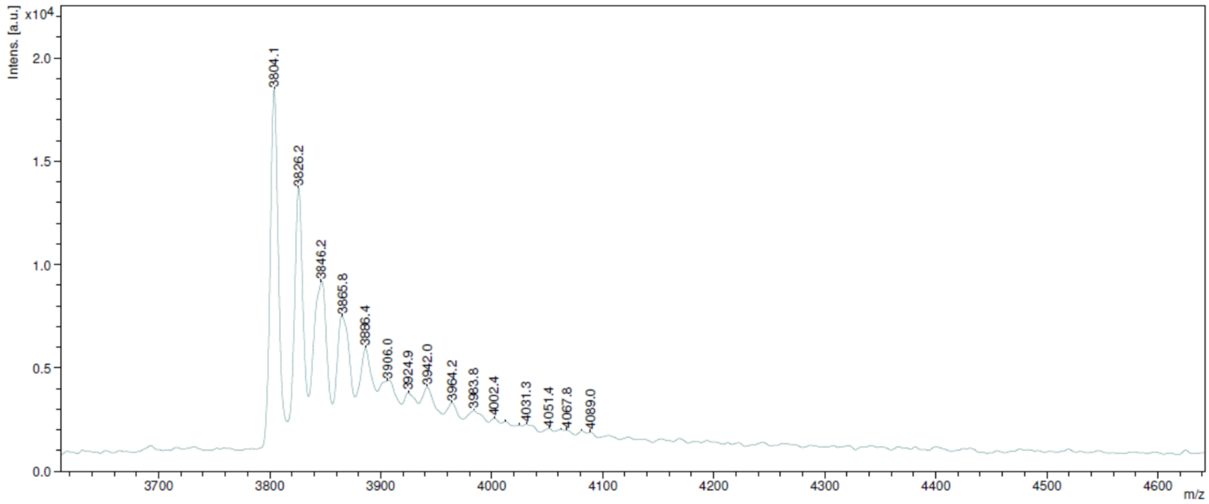

Fig. S17 Mass spectrum of ODN10.

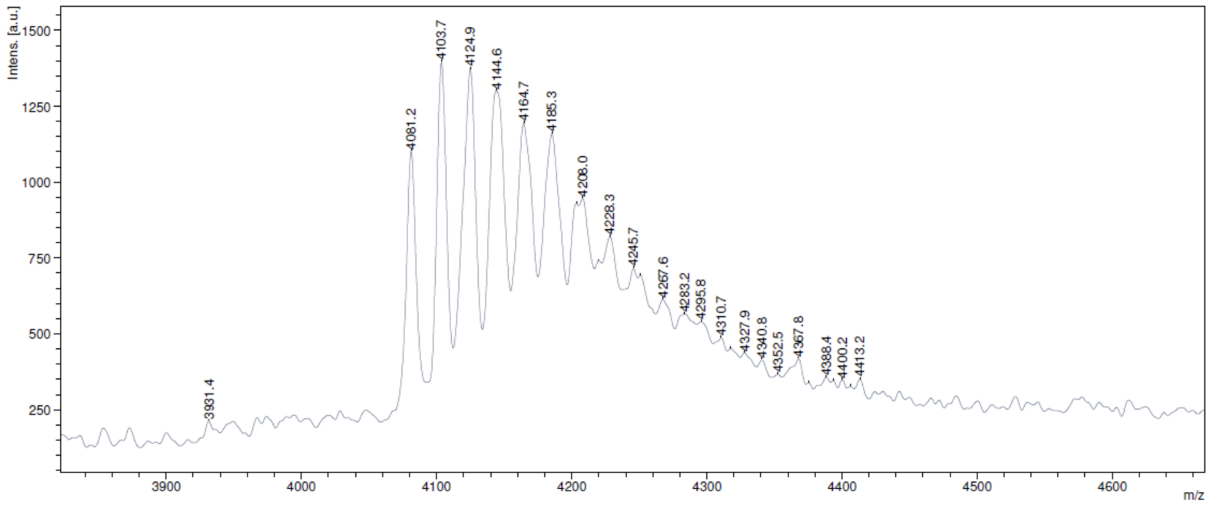

Fig. S18 Mass spectrum of ODN11.

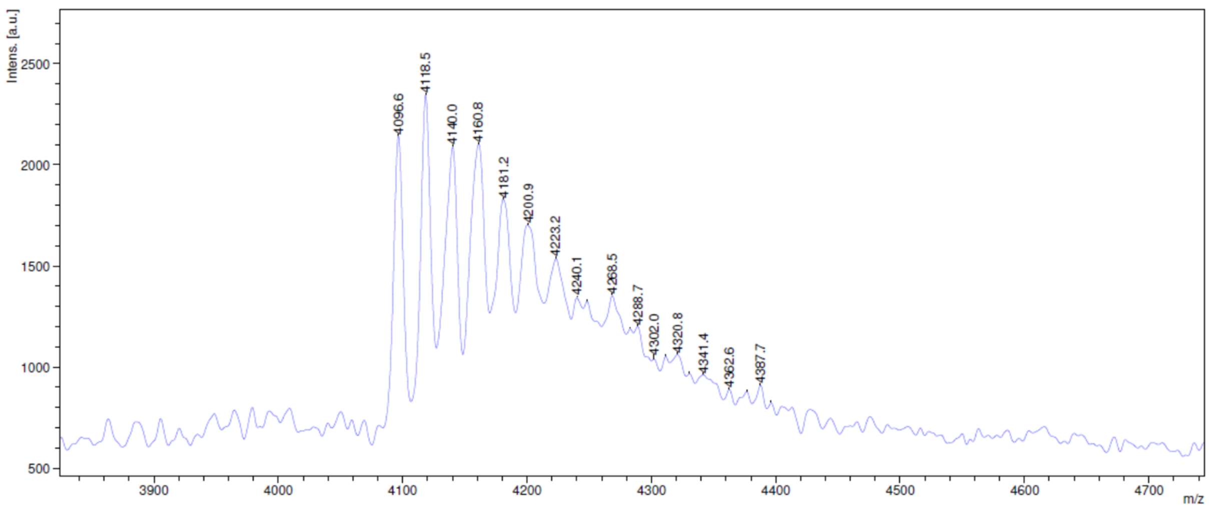

Fig. S19 Mass spectrum of the ODN12.

xyz coordinates of 6PP:C

39

scf done: -1109.837645

C	3.960811	-1.119628	-0.000306
N	2.816983	-0.366644	-0.000308
C	2.869645	0.983794	0.000952
C	4.134628	1.679420	0.002389
C	5.266902	0.919592	0.002413
N	5.208992	-0.451318	0.001113
N	1.710129	1.667939	0.000842
C	6.428938	-1.270642	0.001148
O	3.956464	-2.386626	-0.001426
C	-0.373290	-1.234284	-0.002007
N	-0.669991	0.092660	-0.001243
C	-1.945491	0.505599	-0.000393
C	-2.970611	-0.455691	-0.000302
C	-2.551040	-1.808976	-0.001181
N	-1.270605	-2.233765	-0.002051
N	-4.368789	-0.393538	0.000540
C	-4.760494	-1.663964	0.000094
N	-3.706822	-2.561335	-0.000931
N	-2.202828	1.880334	0.000423
C	-3.439639	2.505980	0.001126
C	-3.197698	3.863359	0.001683
C	-1.771938	3.993588	0.001258
N	-1.162468	2.803479	0.000618
C	-3.783611	-4.022748	-0.001777
H	0.685514	-1.468536	-0.002570
H	-5.787966	-1.992204	0.000526
H	-3.293248	-4.425260	0.887223

H	-4.832314	-4.322829	0.000316
H	-3.297093	-4.424135	-0.893404
H	-1.182161	4.897684	0.001494
H	-3.928028	4.655720	0.002272
H	-4.351019	1.930005	0.001183
H	6.254930	1.363165	0.003450
H	4.184762	2.759252	0.003418
H	0.799526	1.177555	-0.000072
H	1.704593	2.677797	0.001761
H	7.299955	-0.613312	0.002305
H	6.454058	-1.910228	0.886323
H	6.455193	-1.908608	-0.885160

xyz coordinates of ^{7D}6PP:C

40

scf done: -1093.802269

C	4.467012	-0.441607	0.093513
C	3.030840	-0.359889	0.047776
C	2.537682	-1.702120	0.094663
N	3.611276	-2.552141	0.164889
C	4.776772	-1.783148	0.163981
N	1.231846	-2.053038	0.063015
C	0.378682	-1.024053	-0.010443
N	0.733190	0.290912	-0.054349
C	2.034719	0.627047	-0.038252
N	2.328301	1.998939	-0.120722
C	1.453911	3.033950	-0.387074
C	2.195829	4.198663	-0.371796
C	3.535129	3.788035	-0.085509
N	3.622965	2.460442	0.063322
C	3.541422	-4.011143	0.231167
N	-1.813171	1.619028	0.484096
C	-2.945859	0.902088	0.303399
N	-2.833027	-0.354661	-0.166879
C	-3.939204	-1.136782	-0.376740
N	-5.211424	-0.589233	-0.084433
C	-5.328377	0.689394	0.397233
C	-4.232496	1.474597	0.608627
O	-3.874937	-2.321401	-0.816717
C	-6.393070	-1.433539	-0.312816
H	-0.687821	-1.222025	-0.040255
H	5.743516	-2.260883	0.214906
H	2.492857	-4.308580	0.187133

H	4.075210	-4.457409	-0.611934
H	3.976628	-4.373568	1.166324
H	4.418537	4.400214	0.013452
H	1.839695	5.200856	-0.545341
H	0.410748	2.858859	-0.584302
H	-6.332171	1.041733	0.599476
H	-4.329741	2.482202	0.987416
H	-0.894950	1.203364	0.270722
H	-1.861947	2.552335	0.868339
H	-7.289107	-0.875379	-0.036810
H	-6.453001	-1.721357	-1.364831
H	-6.329208	-2.341646	0.290665
H	5.156154	0.383581	0.079689

xyz coordinates of 6PP–Ag(I)–C

40

scf done: -1256.700226

C	-0.017003	0.019006	0.010617
N	0.003803	0.014913	1.388229
N	1.306061	0.008413	1.863306
C	2.083718	0.008606	0.776383
C	1.298440	0.015181	-0.413128
C	-1.079325	0.016430	2.278407
N	-0.802593	0.011478	3.589992
C	-1.804190	0.012481	4.508961
N	-3.114226	0.018205	4.217456
C	-3.381832	0.023225	2.895734
C	-2.413822	0.022711	1.858811
N	-4.605625	0.029649	2.262539
C	-4.345892	0.032744	0.902394
N	-3.047462	0.028776	0.613736
Ag	1.523581	0.000788	4.059930
N	2.451034	-0.008843	5.986116
C	3.784875	-0.014719	6.205014
C	4.303232	-0.020908	7.542316
C	3.403819	-0.020599	8.567821
N	2.052702	-0.014618	8.338197
C	1.545713	-0.008564	7.032716
N	4.616689	-0.014666	5.146327
O	0.302152	-0.003081	6.837996
C	1.081287	-0.014280	9.445792
C	-5.915171	0.032142	2.917411
H	-1.489670	0.008149	5.544633
H	-5.138748	0.037842	0.171261

H	-6.019347	0.920590	3.543689
H	-6.693183	0.038482	2.153153
H	-6.026579	-0.860582	3.536348
H	3.157557	0.004149	0.877402
H	1.654159	0.016812	-1.429784
H	-0.952547	0.024200	-0.526114
H	3.719076	-0.025050	9.603112
H	5.366377	-0.025649	7.732382
H	4.244800	-0.010320	4.203812
H	5.619658	-0.018957	5.269943
H	1.623597	-0.019404	10.391635
H	0.451358	0.875717	9.389554
H	0.444361	-0.898910	9.383953

xyz coordinates of ^{7D}6PP–Ag(I)–C

41

scf done: -1240.662766

N	0.020873	-0.013140	-0.013186
N	-0.002048	0.023866	1.372667
C	1.279828	0.073912	1.869460
C	2.144831	0.066885	0.791613
C	1.311463	0.011547	-0.359941
C	-1.238710	0.013423	2.059404
N	-2.333154	0.029492	1.283645
C	-3.572775	0.025939	1.838514
N	-3.814579	0.010304	3.153432
C	-2.711480	-0.009669	3.932706
C	-1.356793	-0.013695	3.459327
N	-2.690831	-0.037670	5.302562
C	-1.363125	-0.063548	5.718114
C	-0.517033	-0.050516	4.629851
Ag	-1.937890	-0.012075	-1.033632
N	-3.345295	-0.015759	-2.642157
C	-3.024138	-0.029060	-3.955069
C	-4.051149	-0.030239	-4.956222
C	-5.347155	-0.017103	-4.530875
N	-5.665391	-0.003024	-3.197988
C	-4.662916	-0.001845	-2.219734
N	-1.724133	-0.040906	-4.305175
O	-4.971295	0.011807	-0.999454
C	-7.064939	0.013013	-2.738337
C	-3.868597	-0.043576	6.171422
H	-4.401920	0.035757	1.142883
H	-1.117328	-0.091010	6.768627

H	-4.759529	-0.024500	5.542821
H	-3.882957	-0.946739	6.786481
H	-3.867510	0.836560	6.819175
H	1.594337	-0.010072	-1.400467
H	3.220941	0.099070	0.822531
H	1.491861	0.119732	2.921398
H	-6.175986	-0.017054	-5.226821
H	-3.809117	-0.040870	-6.008716
H	-1.003415	-0.039035	-3.593063
H	-1.444493	-0.052032	-5.276196
H	-7.722740	0.004985	-3.607802
H	-7.264666	-0.864201	-2.119784
H	-7.252905	0.910193	-2.145139
H	0.556755	-0.073358	4.697717

xyz coordinates of 6PP–Ag(I)–C × H_2O (I)

43

scf done: -1333.111651

C	0.173672	-0.270299	-0.148292
N	0.155227	-0.131138	1.222564
N	1.441884	-0.138358	1.737494
C	2.250490	-0.283958	0.683185
C	1.499397	-0.371558	-0.525434
C	-0.951288	-0.009969	2.073905
N	-0.712346	0.073943	3.389863
C	-1.739562	0.184816	4.272895
N	-3.039586	0.225671	3.940005
C	-3.268205	0.143389	2.613555
C	-2.271858	0.021497	1.611640
N	-4.472045	0.154023	1.942636
C	-4.173388	0.041273	0.595240
N	-2.868689	-0.041425	0.349754
Ag	1.629126	0.021954	3.934277
N	2.474944	0.002214	5.906314
C	3.793637	-0.063742	6.215343
C	4.205541	-0.221732	7.585538
C	3.238461	-0.270017	8.542917
N	1.908183	-0.172918	8.221872
C	1.502016	-0.036011	6.889522
N	4.712892	0.020193	5.242404
O	0.274751	0.045245	6.615055
C	0.859717	-0.213295	9.255135
C	-5.797753	0.265453	2.553903
H	-1.454948	0.234792	5.315641
H	-4.943733	0.024149	-0.159458

H	-5.888192	1.215811	3.084308
H	-6.555124	0.217804	1.770506
H	-5.955703	-0.555588	3.256312
H	3.317882	-0.312216	0.835314
H	1.883283	-0.490457	-1.524845
H	-0.745108	-0.283695	-0.713013
H	3.476938	-0.381695	9.592686
H	5.251537	-0.291248	7.846403
H	4.491789	0.220766	4.254993
H	5.693022	-0.030171	5.489101
H	1.330470	-0.328709	10.231976
H	0.279292	0.711407	9.235994
H	0.185747	-1.052673	9.072281
O	4.583863	0.802168	2.576324
H	5.457230	0.881817	2.148698
H	4.029260	1.577122	2.367343

xyz coordinates of 6PP–Ag(I)–C × H_2O (II)

43

scf done: -1333.114289

C	0.176355	1.285606	0.525781
N	-0.055934	0.958187	1.847227
N	0.999356	1.360811	2.651615
C	1.877502	1.933712	1.824927
C	1.405695	1.911730	0.477560
C	-1.169963	0.314271	2.397532
N	-1.185870	0.099100	3.723893
C	-2.250367	-0.519491	4.301731
N	-3.339302	-0.959108	3.657513
C	-3.312337	-0.739209	2.327464
C	-2.258120	-0.106838	1.622213
N	-4.258690	-1.060000	1.379569
C	-3.760120	-0.624138	0.163614
N	-2.565570	-0.047381	0.260690
Ag	0.723282	0.878879	4.867720
N	1.952530	-0.045930	6.464974
C	2.722384	-1.130368	6.207936
C	3.503840	-1.735453	7.246292
C	3.441131	-1.177919	8.489360
N	2.662193	-0.077420	8.739549
C	1.906592	0.504247	7.721489
N	2.738908	-1.630132	4.957961
O	1.202830	1.524946	7.995322
C	2.594803	0.530144	10.081275
C	-5.536775	-1.729577	1.629861
H	-2.203901	-0.663336	5.372475
H	-4.307971	-0.752711	-0.756385

H	-6.134495	-1.143385	2.330943
H	-6.078652	-1.823592	0.688034
H	-5.364313	-2.724175	2.046456
H	2.801407	2.335691	2.210125
H	1.903334	2.300972	-0.394921
H	-0.546022	1.043979	-0.237520
H	3.999967	-1.576594	9.325734
H	4.119973	-2.601316	7.053662
H	2.186918	-1.197561	4.226195
H	3.299344	-2.438598	4.725735
H	3.241537	-0.031952	10.755354
H	2.925917	1.569219	10.036243
H	1.567942	0.504971	10.450512
O	-0.109086	2.754749	6.149685
H	0.373547	2.398901	6.959054
H	0.165845	3.657911	5.912181

xyz coordinates of 6PP–Ag(I)–C × H_2O (III)

43			
scf done: -1333.114071			
C	-0.968668	0.566943	3.016392
N	-1.348737	0.566681	4.338691
C	-0.470406	0.877857	5.322206
C	0.875916	1.249361	4.998927
C	1.227457	1.272821	3.681426
N	0.332998	0.940796	2.695400
Ag	-3.435920	0.183761	4.640602
N	-5.121651	0.537208	2.948686
C	-6.363647	0.422752	3.442009
C	-7.458036	0.709802	2.618752
C	-7.141979	1.114765	1.296426
N	-5.897349	1.236420	0.791428
C	-4.925000	0.931205	1.661965
N	-8.843420	0.694768	2.794870
C	-9.331752	1.078162	1.618513
N	-8.350850	1.344256	0.677888
N	-6.498311	0.016409	4.776615
C	-7.654944	-0.223554	5.486377
C	-7.276545	-0.605444	6.759279
C	-5.851899	-0.574547	6.760966
N	-5.380637	-0.200771	5.567550
C	-8.542457	1.783341	-0.705913
N	-0.886283	0.835828	6.599551
C	0.715551	0.947764	1.271188
O	-1.772155	0.236403	2.092598
O	-3.254577	-2.016760	2.130052
H	-3.894646	0.994387	1.337566

H	-10.381079	1.179326	1.390889
H	-8.092622	1.063077	-1.392326
H	-9.611573	1.854061	-0.909684
H	-8.081753	2.761907	-0.856436
H	-5.171426	-0.802641	7.566069
H	-7.924075	-0.869467	7.578444
H	-8.621074	-0.098966	5.023292
H	2.222934	1.548435	3.358997
H	1.586101	1.506695	5.770699
H	-1.836691	0.563516	6.821102
H	-0.261345	1.064368	7.360509
H	1.736960	1.318161	1.183216
H	0.654208	-0.062787	0.862412
H	0.040297	1.596004	0.710373
H	-2.738328	-1.169144	2.101359
H	-4.097452	-1.882482	2.598848

xyz coordinates of 6PP–Ag(I)–C × 2 H_2O (IV)

46

scf done: -1409.531069

C	-0.007701	-0.042779	0.024207
N	-0.043289	-0.000164	1.397111
C	1.102197	0.057333	2.130651
C	2.376822	-0.026286	1.467711
C	2.389019	-0.119992	0.109563
N	1.223599	-0.120953	-0.618337
Ag	-2.026566	-0.253477	2.209474
N	-4.123035	-0.616050	2.694988
C	-4.606514	-1.552861	3.535110
C	-5.992656	-1.730020	3.625533
C	-6.792132	-0.879372	2.823915
N	-6.319742	0.066967	1.989457
C	-4.986908	0.158295	1.959169
N	-6.808890	-2.587573	4.367789
C	-8.047411	-2.254300	4.017162
N	-8.096321	-1.227163	3.088052
N	-3.728084	-2.345593	4.286133
C	-3.967604	-3.617646	4.769856
C	-2.814006	-4.034796	5.403496
C	-1.900285	-2.948204	5.282305
N	-2.447493	-1.927794	4.609113
C	-9.290063	-0.622806	2.492347
N	1.027495	0.192996	3.459602
C	1.233434	-0.184361	-2.090811
O	-1.070578	-0.011656	-0.672782
O	-3.066554	1.565359	0.096854
O	-1.294509	0.466282	4.825355

H	-4.516023	0.867613	1.283561
H	-8.942128	-2.719492	4.399591
H	-9.275077	-0.753804	1.408396
H	-10.175172	-1.111679	2.900873
H	-9.327507	0.442968	2.726928
H	-0.894285	-2.865488	5.663873
H	-2.644828	-4.981198	5.889039
H	-4.920242	-4.098809	4.624359
H	3.310555	-0.187035	-0.453474
H	3.298075	-0.011048	2.031785
H	0.127887	0.327971	3.965376
H	1.882898	0.217230	3.999752
H	2.263197	-0.297175	-2.430889
H	0.639940	-1.035011	-2.430141
H	0.807392	0.730062	-2.508704
H	-2.355421	0.956727	-0.253317
H	-2.722724	2.470006	0.209338
H	-1.844810	-0.376541	4.813517
H	-1.795233	1.255012	5.096540

xyz coordinates of 6PP–Ag(I)–C × 2 H_2O (V)

46

scf done: -1409.530663

C	-0.233215	2.143745	1.225578
N	-0.140374	0.986706	1.972449
N	1.168775	0.757069	2.375994
C	1.872573	1.782947	1.880546
C	1.036724	2.682185	1.155325
C	-1.179584	0.130335	2.353489
N	-1.009287	-0.663585	3.429475
C	-1.992712	-1.541143	3.788750
N	-3.173013	-1.679123	3.178248
C	-3.347055	-0.854737	2.126441
C	-2.389814	0.077502	1.653743
N	-4.431403	-0.738466	1.288416
C	-4.106599	0.237496	0.359984
N	-2.893217	0.752052	0.539766
Ag	0.616498	-0.242347	4.872369
N	2.088665	-0.424593	6.452441
C	3.094881	-1.340299	6.442575
C	3.918873	-1.509284	7.610046
C	3.680566	-0.707271	8.684064
N	2.689499	0.243571	8.660676
C	1.885062	0.396894	7.534356
N	3.310946	-2.074797	5.344136
O	0.985376	1.293938	7.543282
C	2.447079	1.133022	9.810441
C	-5.676880	-1.504251	1.376157
H	-1.790085	-2.167614	4.645978
H	-4.790758	0.531497	-0.420150

H	-6.168766	-1.311389	2.331731
H	-6.337416	-1.199423	0.563772
H	-5.466218	-2.571889	1.286896
H	2.937897	1.834950	2.044394
H	1.331580	3.588912	0.654238
H	-1.176095	2.467606	0.816302
H	4.262144	-0.777164	9.593852
H	4.708408	-2.246388	7.630413
H	2.802694	-1.935564	4.448338
H	4.059563	-2.756038	5.352117
H	3.145705	0.876300	10.607222
H	2.594390	2.173773	9.515433
H	1.422776	1.011267	10.167326
O	-0.249894	2.104556	5.384789
H	0.193561	1.912659	6.264129
H	0.194218	2.825853	4.903138
O	2.179278	-1.762319	2.861341
H	1.619291	-2.473798	2.501490
H	1.817440	-0.860818	2.624272

xyz coordinates of H_2O

3

scf done: -76.392468

O	0.000000	0.000000	0.000000
H	0.000000	0.000000	0.942036
H	0.913426	0.000000	-0.230505
